# Persistent serum protein signatures define an inflammatory subcategory of long COVID

**DOI:** 10.1038/s41467-023-38682-4

**Published:** 2023-06-09

**Authors:** Aarthi Talla, Suhas V. Vasaikar, Gregory Lee Szeto, Maria P. Lemos, Julie L. Czartoski, Hugh MacMillan, Zoe Moodie, Kristen W. Cohen, Lamar B. Fleming, Zachary Thomson, Lauren Okada, Lynne A. Becker, Ernest M. Coffey, Stephen C. De Rosa, Evan W. Newell, Peter J. Skene, Xiaojun Li, Thomas F. Bumol, M. Juliana McElrath, Troy R. Torgerson

**Affiliations:** 1 Allen Institute for Immunology, Seattle, WA USA; 2 Vaccine and Infectious Disease Division, Fred Hutchinson Cancer Center, Seattle, WA USA; 3 Present Address: Seagen, Bothell, WA USA; 4 Present Address: Moderna, Cambridge, MA USA

**Keywords:** SARS-CoV-2, Chronic inflammation, Cytokines, Chemokines

## Abstract

Long COVID or post-acute sequelae of SARS-CoV-2 (PASC) is a clinical syndrome featuring diverse symptoms that can persist for months following acute SARS-CoV-2 infection. The aetiologies may include persistent inflammation, unresolved tissue damage or delayed clearance of viral protein or RNA, but the biological differences they represent are not fully understood. Here we evaluate the serum proteome in samples, longitudinally collected from 55 PASC individuals with symptoms lasting ≥60 days after onset of acute infection, in comparison to samples from symptomatically recovered SARS-CoV-2 infected and uninfected individuals. Our analysis indicates heterogeneity in PASC and identified subsets with distinct signatures of persistent inflammation. Type II interferon signaling and canonical NF-κB signaling (particularly associated with TNF), appear to be the most differentially enriched signaling pathways, distinguishing a group of patients characterized also by a persistent neutrophil activation signature. These findings help to clarify biological diversity within PASC, identify participants with molecular evidence of persistent inflammation, and highlight dominant pathways that may have diagnostic or therapeutic relevance, including a protein panel that we propose as having diagnostic utility for differentiating inflammatory and non-inflammatory PASC.

## Abstract (web summary)

Long COVID or post-acute sequelae of SARS-CoV-2 is defined by persisting chronic symptoms following acute SARS-CoV-2 infection but represent an aetiologically diverse group of disorders. Here authors identify molecularly distinct subtypes, including a form with persistent inflammation, via longitudinal analysis of serum proteome.

## Introduction

New, recurrent, or prolonged symptoms after acute SARS-CoV-2 infection are termed post-acute sequelae of SARS-CoV-2 (PASC) or long COVID. A systematic review of 38 papers reported that one-third or more of surviving COVID-19 participants experienced at least one PASC symptom during the 2–5 months after the onset of acute infection^1^. PASC symptoms are numerous and varied, impacting virtually every major organ system (https://www.cdc.gov/coronavirus/2019-ncov/long-term-effects/index.html)^2,3^ and can last for weeks or months. Recent Delphi consensus criteria propose that PASC be defined as having at least 60 days of symptoms persisting for a minimum of 90 days post-symptom onset (https://www.who.int/publications/i/item/WHO-2019-nCoV-Post_COVID-19_condition-Clinical_case_definition-2021.1). However, the published literature is inconsistent, defining PASC as persistent symptoms for 30–120 days post-symptom onset^4–7^. Despite the large number of individuals affected, the lack of consensus diagnostic criteria or standardized outcome measures impede efforts to effectively group persons to establish clinical etiologies or to evaluate outcomes of therapeutic trials^8^. There are a limited number of clearly defined and validated molecular markers of disease^9,10^ or definitive diagnostic tests. To make matters more complicated, it is recognized that similar clinical symptoms could arise after acute infection regardless of whether they were caused by persistent inflammatory disease initiated by the viral immune response, unresolved organ or tissue damage, or delayed viral clearance. Identification of molecular features capable of mechanistically defining the heterogeneity of PASC could be transformative, allowing clinicians and researchers to better subset participants for clinical trials and highlighting potential targets for therapeutic intervention.

Here, we use the Olink proteomics platform to analyze the serum proteome of 55 unvaccinated adults with PASC who were infected with the ancestral strain of SARS-CoV-2. We show that a subset of PASC patients have evidence of persistent inflammation. Among those with persistent inflammation, individuals with PASC, cluster into two groups: One, dominated by increased Interferon-γ, a signature of type II IFN-driven inflammation, NF-κB activation, and increased inflammatory cytokines, chemokines, and cytokine receptors. The second, dominated by a signature of persistent neutrophil activation, NF-κB activation, and type I IFN-driven inflammation. The findings suggest that the identified inflammatory serum protein signature could be used to stratify patients for clinical trials of immunomodulatory drugs to identify individuals that may benefit most from treatment. The signature or the component proteins may also be useful biomarkers to evaluate therapeutic responses.

## Results

### Reported symptoms are diverse and unable to differentiate subsets of PASC in our cohort

The study cohort consisted of 55 adults (21 men, 34 women; age 22–82 years) with persistent symptoms lasting ≥60 days after an acute, PCR-confirmed SARS-CoV-2 infection (termed “PASC”), 24 (9 men, 15 women; age 20–79 years) who symptomatically recovered after a PCR-confirmed SARS-CoV-2 infection (termed “Recovered”), and 22 (12 men, 10 women; age 29–77) who had a negative nasopharyngeal PCR test (termed “Uninfected”). Participants were enrolled during the ancestral strain infection of the COVID-19 pandemic. The majority of participants had mild COVID symptoms during their acute SARS-CoV-2 infection (World Health Organization (WHO) ordinal scale 2 or 3)^11^. Only 3 participants were hospitalized and required oxygen therapy (WHO ordinal scale 5). Two of these received Remdesivir. One additional participant received steroids. No participants in this cohort required mechanical ventilation or underwent chest computed tomography (CT). All were unvaccinated when enrolled in the study. Summary demographics for the cohort are shown in supplemental Table S1. The uninfected individuals had blood drawn once at study entry while the PASC and recovered participants had one or more blood draws at timepoints ≥60 days and up to 379 days post-symptom onset (PSO) of acute COVID (see Supplemental Fig. S1). Symptoms and other clinical metadata for each PASC participant are provided in supplemental Data S1. Previous studies have divided PASC participants into subsets based on either type, number, or severity of clinical features^7,12–14^. PASC participants in our cohort reported multiple symptoms at ≥60 days PSO ranging from fatigue, fever, chills to more clinically concerning symptoms like arrhythmia or brain fog. These individual PASC symptoms were combined into organ-related symptom groups like pulmonary, cardiovascular, neurologic, etc. (see Methods) (Supplemental Data S1). For our cohort, hierarchical clustering on PASC symptomatology alone at ≥60 days PSO did not clearly drive significant participant clustering (Supplemental Table S1 & Fig. S2A). We next attempted to cluster based on serum proteins that were significantly associated with reported symptoms, but no single symptom or combination of symptoms was able to clearly distinguish participant groups (Supplemental Fig. S2B–D) suggesting that symptoms alone are unable to differentiate subsets of PASC and that additional biologic measures are needed to clarify the groups.

### Clustering of the serum proteome using an unsupervised learning approach identifies a subset of PASC participants that have evidence of persistent inflammation

We analyzed the serum proteome using the Olink Explore 1536 panel (see Methods, supplemental Data S2) to first interrogate specific proteins that distinguish PASC (at their first time point available ≥60-days post symptom onset), recovered (at their last time point available ≥60-days post symptom onset), and uninfected participants in our cohort. We identified 275, 25, and 14 proteins that were significantly differentially expressed (*p* < 0.05) between PASC, recovered, and uninfected groups respectively when each group was compared to the other groups (Supplemental Data S3). We noted that within the PASC group, there was variation in expression of the serum proteomic signature, suggesting that some have an inflammatory signature and others do not (Supplemental Fig. S2E), and highlighting the heterogeneous nature of the disorder.

**Fig. 1:**
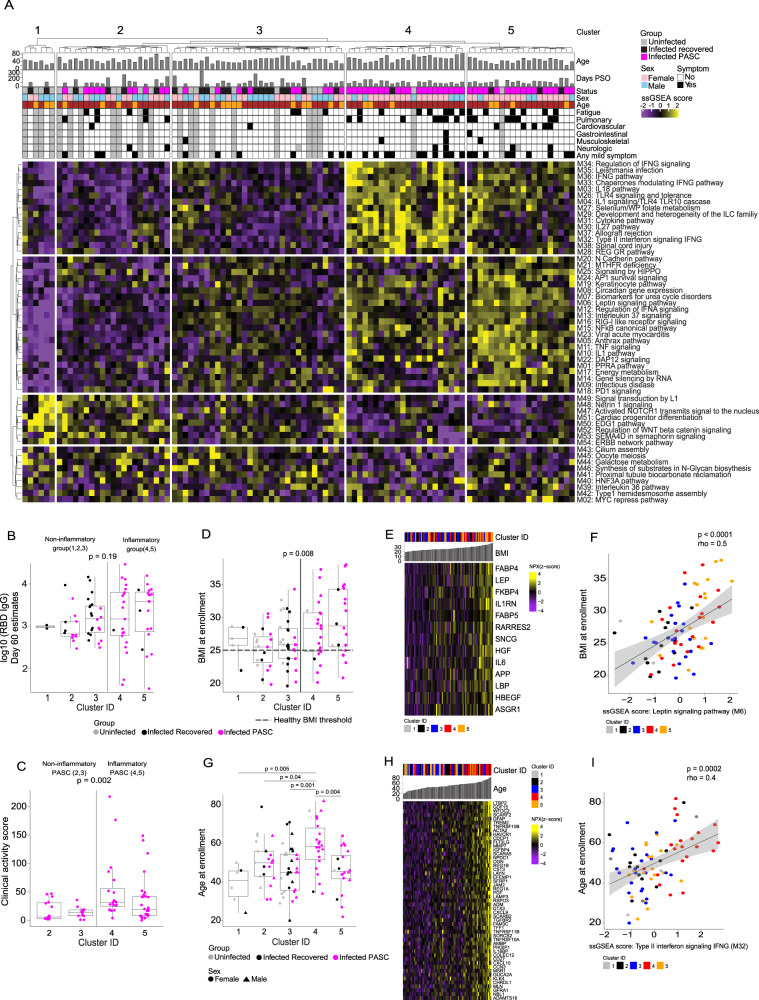
Serum proteomic clustering and clinical metadata of PASC. **A** Single Sample Gene Set Enrichment Analysis (ssGSEA) score heatmap of the rule-in selected serum proteome modules (rows), across 55 PASC, 24 recovered and 22 uninfected participants (columns). **B** Receptor binding domain (RBD)-specific IgG titers (y-axis) in 55 PASC and 24 recovered participants between clusters. The *p*-value was calculated comparing, as a group, inflammatory versus non-inflammatory clusters using a two-sided Wilcoxon test. **C** Clinical activity score (y-axis) of acute COVID symptoms in 55 PASC participants from inflammatory (4 & 5) vs. non-inflammatory (2 & 3) clusters. The *p*-value was calculated by comparing inflammatory PASC versus non-inflammatory PASC using a two-sided Wilcoxon test. **D** Body Mass Index (BMI) at enrollment (y-axis) across clusters (x-axis) in 55 PASC, 24 recovered and 22 uninfected. Healthy BMI cutoff is indicated by the dashed line. The *p*-value was calculated by comparing inflammatory clusters (4,5) to the other clusters using a two-sided Wilcoxon test. **E** Heatmap of proteins (rows) significantly (*p*-value < 0.05) correlated with BMI across all COVID-19+ participants (columns). The *p*-values were determined by a two-sided Spearman’s correlation test. **F** Correlation between the ssGSEA score of the leptin signaling module (x-axis) and BMI at enrollment (y-axis) across all COVID-19+ participants. The *p*-value was determined by a two-sided Spearman’s correlation test. **G** The age at enrollment (y-axis) across clusters (x-axis), in 55 PASC, 24 recovered and 22 uninfected. *P*-values were determined by a two-sided Wilcoxon test by pairwise cluster comparison. Box plots show the median (centerline), the first and third quartiles (the lower and upper bound of the box) and the whiskers show the 1.5x interquartile range of the data. **H** Heatmap of the proteins (as rows) significantly (*p*-value < 0.05) correlated with age across all COVID-19+ participants (as columns). The *p*-values were determined by a two-sided Spearman’s correlation test. **i** Correlation between the ssGSEA score of the type II interferon signaling module (x-axis) and age at enrollment (y-axis) across all COVID-19+ participants. The *p*-value was determined by a two-sided Spearman’s correlation test. The bands in all correlation scatter plots display the 95% confidence interval.

We hence took an alternative approach, using unbiased clustering of the serum proteome across the entire cohort (PASC+recovered +uninfected) to find clusters of individuals that had similar serum proteome signatures regardless of their COVID-19 status or reported symptomatology. For this purpose, we used the first ≥60-day sample available for each PASC participant, the last available post ≥60-day sample for each recovered participant (to maximize the chance that proteome alterations had returned to baseline), and the solitary sample from uninfected individuals (see Methods for details). We used curated canonical pathways from the Molecular Signatures Database (MSigDB) and applied a rule-in statistical approach (see Methods) to identify pathways that distinguished PASC from both recovered and uninfected individuals^15^. This resulted in identification of 85 pathways that have a significant rule-in performance (*p* < 0.01). Since MSigDB is a collection of annotated pathways generated from several different experimental datasets, pathways may contain overlapping genes/gene products. To avoid redundancy of genes and gene sets, the identified pathways were merged into 54 modules using the enrichment map approach with a minimum Jaccard index threshold of 25% (see Supplemental Data S4 and Methods)^16^. Hierarchical k-means clustering using the 54 proteomic modules identified 5 discrete clusters that showed distinct expression patterns of the modules (Fig. 1A). Two of the clusters (4 & 5) showed marked enrichment for inflammatory modules including type I and type II interferon signaling, TNF signaling, NFκB signaling, and several others, while clusters 1, 2, and 3 lacked a distinct inflammatory protein signature. Inflammatory clusters 4 and 5 included predominantly PASC (91% and 80% respectively). In contrast, cluster 1 consisted of only uninfected or recovered participants and clusters 2 and 3 consisted of a mixture of recovered, uninfected, and PASC with a lower percentage of participants being from the PASC group (48% and 28% respectively) (Supplemental Fig. S3A). The distribution of PASC participants across inflammatory (4 & 5; 65% of PASC) and non-inflammatory (2 & 3; 35% of PASC) proteomic clusters underscores the heterogeneity of PASC and was our first clear indication that only a subset of PASC participants have ongoing inflammation. To assess if the differential serum proteomic signatures captured at the initial post-60 day time point persisted over time, we extended our analysis to include all longitudinal samples available for each participant. We found that PASC participants exhibiting an inflammatory protein signature continue to have that signature over time and that most participants remained in the same cluster throughout the longitudinal study period (Supplemental Fig. S3B). It is unclear whether the two clusters of inflammatory PASC (4 & 5) are unique with distinct molecular drivers or represent a spectrum of disease.

It is possible that a poor immune response to SARS-CoV-2 may allow delayed clearance of viral particles that could increase the risk for developing PASC with persistent inflammation. To address this question, we evaluated SARS-CoV-2 receptor binding domain (RBD)-specific IgG titers in serum samples obtained 60 days PSO from PASC and recovered participants in each of the 5 clusters identified above.

There was no significant difference in RBD-specific IgG responses between previously infected COVID participants in any of the clusters (Fig. 1B). We also compared SARS-CoV-2-specific CD4+ and CD8+T cell frequencies^17,18^ between PASC and recovered participants from all clusters and did not identify any significant differences (Supplemental Fig. S3C & S3D).

### More severe acute COVID symptoms correlate with an increased likelihood of developing persistent inflammation in the PASC period

Previous studies have suggested that severity of the acute infection may lead to a higher incidence of PASC^19–21^. We hypothesized that an inflammatory serum protein signature may also correlate with being more symptomatic during acute infection. However, because our cohort primarily experienced mild acute COVID-19 symptoms (WHO ordinal scale 2 or 3), commonly used COVID severity indices did not capture the range of heterogeneity in symptomatology that we observed clinically. We therefore developed a clinical activity score for the acute phase of mild COVID. The clinical activity score for each participant reflects the impact on Activities of Daily Living (ADLs) for each day of illness. The score evaluates symptom activity for each day of illness ranging from no symptoms to life threatening illness with profound effect on ADLs and accounts for the time spent at each level (see Methods). Inflammatory PASC participants in clusters 4 & 5 had a significantly higher pre-PASC clinical activity score or higher impact on ADLs (two-sided Wilcoxon test *p* = 0.002) compared to non-inflammatory PASC participants in clusters 2 & 3 (Fig. 1C).

### Higher BMI and older age are associated with having persistent inflammation in PASC

We assessed if other covariates or clinical parameters such as age, gender, BMI, or underlying comorbidities were associated with having an inflammatory serum proteomic signature. While there were no overall significant differences in age or BMI at enrollment between PASC, recovered and uninfected participants (Supplemental Figs. S4A & S4B), BMI was significantly higher in the inflammatory PASC groups (clusters 4 and 5) compared to participants in the other non-inflammatory clusters (Fig. 1D). We noted that well-described BMI associated proteins like (Leptin (LEP) and Fatty Acid Binding Protein (FABP4)) and the Leptin signaling module were significantly increased in participants with high BMI (clusters 4 and 5) in our cohort (Fig. 1E, F)^22–24^. We also noted that interleukin-6 (IL-6), identified as part of the inflammatory signature associated with PASC, was positively correlated with BMI (Fig. 1E). These data suggest that BMI may be a risk factor for exhibiting an inflammatory PASC phenotype but does not account fully for the increased IL-6 observed in inflammatory PASC because a small subset of participants whose BMI was within the “healthy” range also had increased IL-6 as part of their inflammatory proteomic signature.

We next evaluated the impact of age on the inflammatory serum protein signature because we noted that PASC participants in cluster 4 were significantly older (Fig. 1G). Specifically, CXCL9, CXCL10, and IL18BP in addition to the type II interferon signaling module that have been associated with the process described as “inflammaging”^25^, were positively correlated with higher age in cluster 4 in our cohort (Fig. 1H, I). Inflammatory proteins present in the published proteome data associated with obesity or age^22–24,26^ overlapped with some of the signature proteins we found associated with inflammatory PASC but did not account for the breadth of the signature we identified. We have summarized the numerical overlap of protein markers reported in obesity, aging, and the PASC inflammatory signature in a Venn diagram (Supplemental Fig. S4C, Data S5) to demonstrate the similarities and differences between these states. Together, these data suggest that increased BMI and older age may be associated with a risk for persistent inflammation in participants with PASC but do not account for the full inflammatory signature observed in inflammatory PASC since several identified inflammatory markers were specific to PASC. We also noted a non-significant (*p* = 0.088) trend toward higher blood pressure in the inflammatory clusters (4 & 5) of PASC participants (Supplemental Fig. S4D) but the known association between increased blood pressure, high BMI, and older age confounds interpretation of this finding. Very few participants had other comorbidities and the presence or absence of other comorbidities was not significantly associated with the inflammatory serum proteomic signature. All p-values tested for the presence or absence of a comorbidity between the inflammatory and non-inflammatory groups with a Fisher Exact test were >0.05 (Fig. S4D). While these results are interesting, additional larger studies will be needed to confirm these observations.

### Signaling modules and specific inflammatory proteins that define the inflammatory form of PASC

**Fig. 2:**
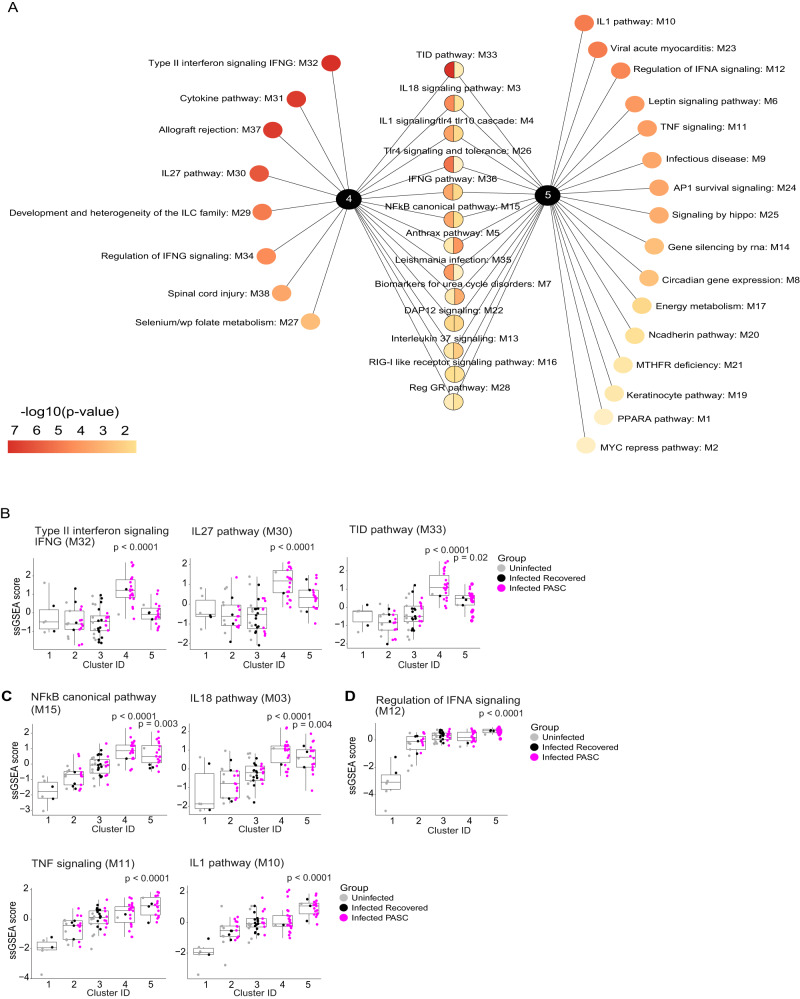
Key pathway modules driving inflammatory PASC signatures. **A** Modules that are significantly expressed more highly in clusters 4 and 5 relative to all other clusters. Modules unique to a cluster are arranged and ranked by increasing adjusted *p*-value of <0.05, while modules expressed in both clusters are arranged and ranked by the average of their adjusted *p*-values. The color gradient of each node represents the -log10 adjusted *p*-value. *P*-values were determined by a two-sided Wilcoxon test. **B**–**D** Box and jitter plots of the Single Sample Gene Set Enrichment Analysis (ssGSEA) scores (y-axis) across all clusters (x-axis) (that consist of PASC, *n* = 55; recovered, *n* = 24; uninfected, *n* = 22 participants) for the top-ranked modules that were enriched in inflammatory clusters 4 and 5. *P*-values determined by a two-sided Wilcoxon test were calculated comparing inflammatory cluster 4 and inflammatory cluster 5 independently to clusters 1,2,3. Box plots show the median (centerline), the first and third quartiles (the lower and upper bound of the box) and the whiskers show the 1.5x interquartile range of the data.

Among the 54 modules that defined the 5 clusters of participants (Fig. 1A), we identified those that significantly distinguished each cluster by calculating the single-sample Gene Set Enrichment Analysis (ssGSEA) score per module across samples (Supplemental Data S6)^27^. Ranking modules by adjusted *p*-value identified those most significantly associated with inflammatory clusters 4 and 5 (Fig. 2A and Supplemental Data S7 & Fig. S5). Within cluster 4, multiple pathways associated with type II interferon (IFN-γ) signaling (Type II IFN signaling, IL-27, TID (aka: Chaperones modulating interferon signaling)), were among those most highly enriched (Fig. 2B, Supplemental Data S7). Canonical NF-κB signaling and NF-κB activating cytokine pathways (IL-18, TNF, IL-1 were enriched in both clusters 4 and 5 (Fig. 2C). In addition, cluster 5 was also enriched for proteins associated with IFN-α signaling (Fig. 2D). The expression scores of these modules across all samples were significantly correlated with each other, indicating, participants with higher IFN-γ signaling have higher IL-27, IL-18, and NF-κB signaling, and participants with higher TNF signaling have higher IL-1, NF-κB, and IFN-α signaling, suggesting a coordinated activation of immune cascades that drive inflammation (Supplemental Fig. S6).

**Fig. 3:**
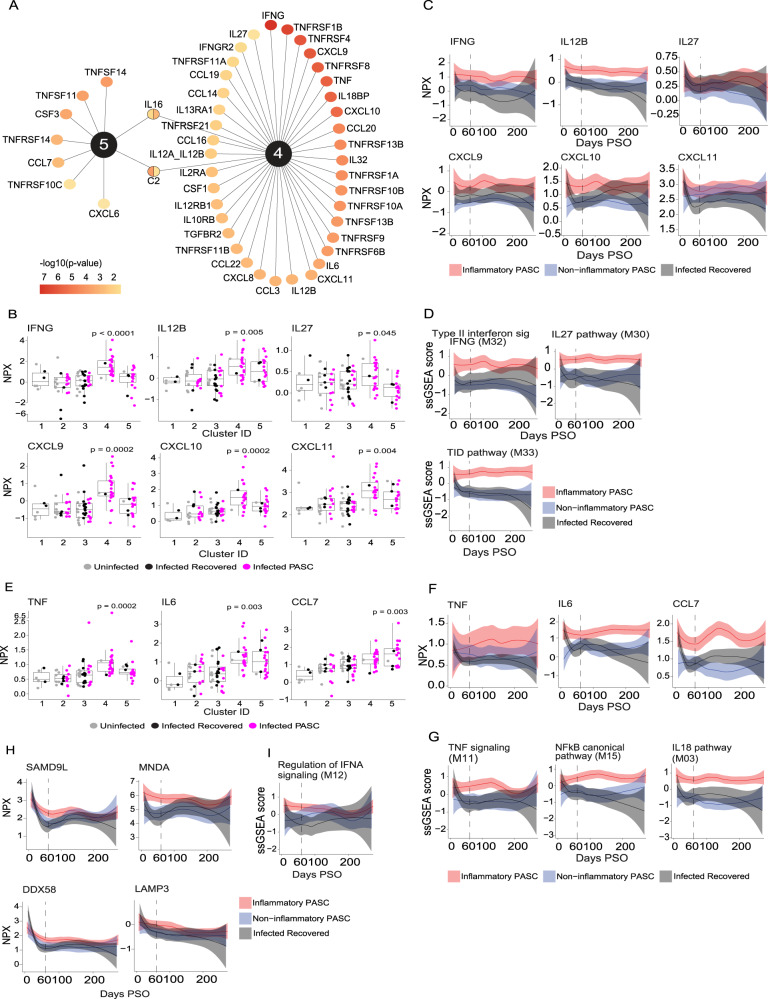
Longitudinal assessment of key cytokines and chemokines driving inflammatory PASC signatures. **A** Differentially expressed cytokines, chemokines, and their receptors up-regulated in inflammatory clusters 4 & 5. Proteins significantly up-regulated in clusters 4 and 5 relative to all other clusters are reported. *P*-values were tested by a two-sided Wilcoxon test and adjusted for multiple comparisons. The color gradient of nodes represents the -log10 adjusted *p*-value. **B** Box plots of IFN-γ Normalized Protein Expression (NPX) (y-axis) and its related cytokines and chemokines across clusters (x-axis) in 55 PASC, 24 recovered, 22 uninfected participants which were significantly upregulated exclusively in cluster 4. *P*-values were calculated comparing inflammatory clusters 4 and 5 independently to clusters 1,2,3, using a two-sided Wilcoxon test. **C** Longitudinal Loess fit plots of IFN-γ NPX (y-axis) and its related cytokines and chemokines on samples available from early acute infection through >250 days post symptom onset (PSO) (x-axis). PASC participants from inflammatory clusters 4 and 5 are represented as inflammatory PASC (red), PASC participants from clusters 2 and 3 as non-inflammatory PASC (blue) while the recovered participants are in black. **D** Longitudinal Loess fit plots of the Single Sample Gene Set Enrichment Analysis (ssGSEA) scores (y-axis) of IFN-γ related modules over time (x-axis). **E** Box plots of TNF, IL6 and CCL7 NPX (y-axis) across clusters (x-axis) in 55 PASC, 24 recovered, 22 uninfected participants which were significantly upregulated in clusters 4 and 5. *P*-values were calculated comparing clusters 4 and 5 independently to clusters 1,2,3, using a two-sided Wilcoxon test. All box plots show the median (centerline), the first and third quartiles (the lower and upper bound of the box) and the whiskers show the 1.5x interquartile range of the data. **F** Longitudinal Loess fit plots of TNF, IL6 and CCL7 NPX (y-axis) over time (x-axis). **G** Longitudinal Loess fit plots of the ssGSEA scores (y-axis) of TNF and NF-κB related signaling modules over time (x-axis). **H**, **I** Longitudinal Loess fit plots of NPX and ssGSEA scores (y-axes) of type-I IFN-driven proteins and the IFN-α module over time (x-axis) respectively. The bands in all loess smooth fit plots display the 95% confidence interval.

We next investigated the individual proteins differentially expressed in the serum of participants within each cluster. Each cluster (1-5) was individually compared to all other clusters. Cluster 4 had 234 differentially expressed proteins (DEPs) whereas cluster 5 had 296 DEPs (Supplemental Data S8; adj. *p*-value < 0.05, Supplemental Fig. S7). Based on our initial findings that the dominant modules in these two inflammatory clusters were associated with cytokines, chemokines, their receptors and associated signaling pathways, we focused first on these proteins since they are major drivers of inflammation and potential targets for therapeutic intervention. Differentially expressed cytokines, chemokines, and cytokine/chemokine receptors were ranked by adjusted p-value. IFN-γ was the cytokine that most significantly defines cluster 4 (Fig. 3A, B, Supplemental Fig. S8, Supplemental Data S8). In addition to IFN-γ, increased expression of chemokines and cytokines known to be regulated by IFN-γ including CXCL9, CXCL10, CXCL11 and IL-27 in cluster 4 suggests that it is functionally active. We also observed increased expression of IL-12 p40 (IL12B) and the IL-12 p40/p70 heterodimer (IL12A_IL12B) in cluster 4, which may drive expression of IFN-γ and an overall Th1 signature (Fig. 3A, B). While not as strongly differentially expressed in cluster 5, IFN-γ and IFN-γ induced chemokines are enriched compared to those that recovered from COVID or were uninfected (Fig. 3B).

To determine whether IFN-γ and IFN-γ driven cytokines and chemokines remained persistently elevated over time in inflammatory PASC, we compared expression levels in all inflammatory PASC individuals (clusters 4 + 5) with those observed in non-inflammatory PASC or in individuals that were infected with SARS-CoV-2 but recovered. Proteins were evaluated longitudinally in all available samples beginning from early acute infection to 275 days post-symptom onset (PSO). IFN-γ, IL-12 p40, and IFN-γ-driven chemokines were consistently elevated in inflammatory PASC relative to the other 2 groups (Fig. 3C and supplemental Fig. S9). To strengthen this observation, we extended our analysis to include all proteins that are part of IFN-γ related signaling modules and show that over time, the differences between inflammatory PASC vs. non-inflammatory PASC and the recovered COVID groups are even more distinct (Fig. 3D and Supplemental Fig. S10A).

In addition to the IFN-γ related signature noted above, we also observed that TNF, TNF-driven cytokines and chemokines (including IL-6 and CCL7 (MCP3)), and several TNF receptor superfamily members were also increased in both inflammatory PASC clusters but most extensively in cluster 4 (Fig. 3A, E). TNF, IL-6, and CCL7 remained persistently elevated in all inflammatory PASC (clusters 4 + 5) over time when compared to non-inflammatory PASC or individuals who were infected but recovered (Fig. 3F). In addition to elevated cytokine levels, there is evidence for persistent inflammatory cytokine signaling based on the enrichment of proteins involved in TNF signaling, the IL-18 pathway, and the NF-κB canonical signaling pathway (Fig. 3G and Supplemental Fig. S10B).

In inflammatory PASC cluster 5, we also noted a protein signature suggestive of persistent type I interferon signaling including elevation of proteins that are induced by type I IFNs during acute SARS-CoV-2 infection (SAMD9L, DDX58, MNDA, and LAMP3)^28,29^. Interestingly, these type I IFN associated proteins were increased at the earliest sampling timepoint available for analysis in inflammatory PASC and remained elevated for approximately 180 days post infection (Fig. 3H and Supplemental Figs. S8 & S9). Similarly, ssGSEA analysis showed enrichment for the pathway associated with regulation of IFN-α signaling in inflammatory PASC that followed a similar kinetic (Fig. 3I and supplemental Fig. S10C). We do not have a specific readout for systemic type I IFN levels since these are notoriously difficult to accurately quantify in circulation and the Olink assay only measures IFN-γ and IFN-λ1, however the accumulated evidence points to the persistent activity of type-I interferons in PASC individuals that exhibit signs of persistent inflammation. This is notable considering recent studies reporting detection of SARS-CoV-2 RNA and spike protein in gastrointestinal and hepatic tissue of convalescent participants up to 180 days after acute infection, diverse extrapulmonary tissues including brain up to 230 days after acute symptom onset, and SARS-CoV-2 spike protein in serum up to a year post-acute infection^30–32^. Whether residual viral RNA and/or protein may serve as a driver of the phenotype in inflammatory PASC remains to be investigated more thoroughly.

**Fig. 4:**
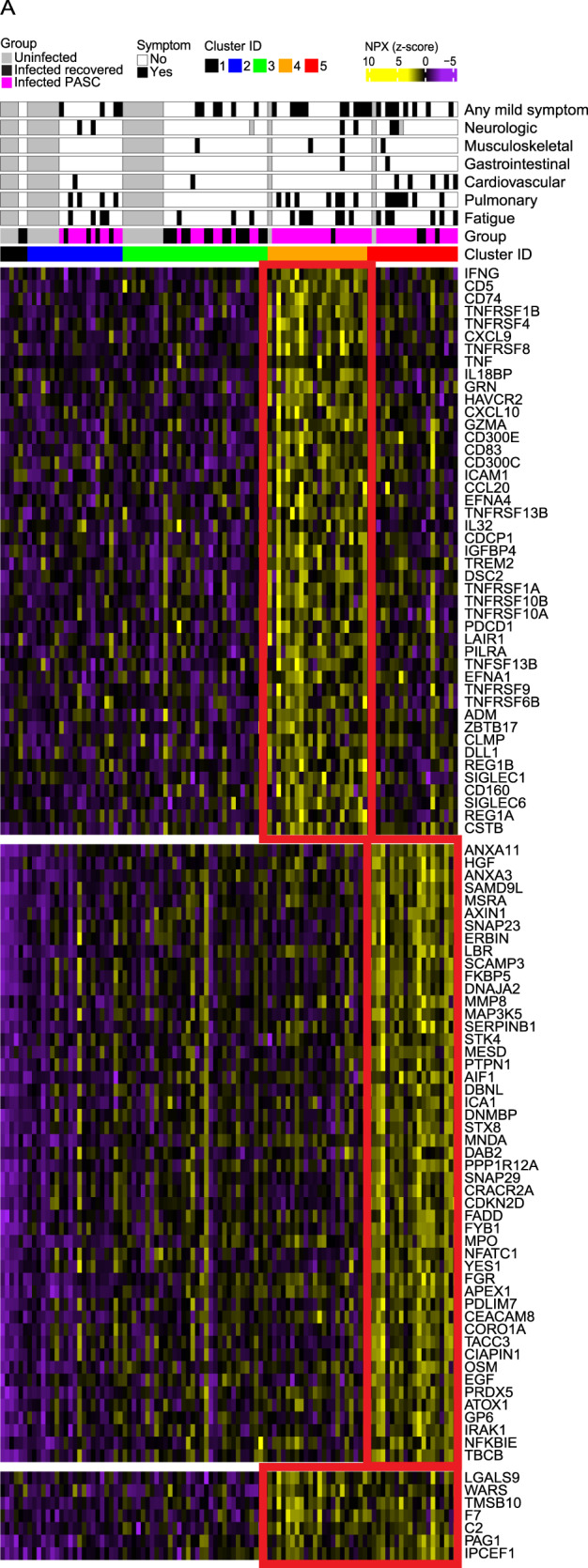
Top 50 overall serum proteins driving the signatures observed in the two inflammatory PASC clusters. **A** Heatmap of the top 50 Olink serum proteins ranked by adjusted *p*-value < 0.05 that are up-regulated in inflammatory clusters 4 and 5 compared to all other clusters. Rows represent individual proteins; columns represent individual samples and the scaled Normalized Protein Expression (NPX) expression across samples is depicted from low (purple) to high (yellow). The *p*-value determined by a two-sided Wilcoxon test was calculated comparing one cluster of participants to all other groups.

To enhance our understanding of broader immune events that may be occurring in PASC individuals with persistent inflammation, we expanded our analysis beyond cytokines, chemokines, and their receptors to focus on the top 50 proteins differentially expressed in inflammatory PASC clusters 4 & 5 versus the non-inflammatory clusters (Fig. 4A). Two important observations emerge from this analysis: First, IFN-γ is both the most highly expressed cytokine in cluster 4 and the most highly differentially expressed protein overall in this cluster. Similarly, many of the top 50 most differentially expressed proteins in cluster 4 are the cytokines, chemokines, or cytokine/chemokine receptor subunits highlighted above (Fig. 4A, upper portion). Cluster 4 participants also showed increased levels of cell surface receptors linked to inflammation (CD74) or inflammation-associated checkpoint molecules (CD5, TIM-3 (HAVCR2), PDCD1, and CD83). Second, we noted that in cluster 5, cytokines, chemokines, and their receptors were less prominent but two of the three most highly differentially expressed proteins overall were annexin-11 (ANXA11) and annexin-3 (ANXA3) (Fig. 4A, lower portion). Among annexins, ANXA3 is most highly restricted to neutrophils and ANXA11 is also strongly expressed in this cell type^33^. ANXA11 along with ANXA3 translocate to the neutrophil granule membrane in a calcium-dependent manner and are released upon degranulation^34^. Elevated peripheral blood ANXA3 levels have been correlated with inflammatory diseases like sepsis where it has been associated with poor outcomes or death and stimulation of whole blood with various toll-like receptor agonists, fixed pathogens, and cytokines showed that ANXA3 was upregulated most strongly by IFN-γ but also induced by IFN-β and poly-IC^33^. Pairing this with the presence of neutrophil granule proteins including MMP8 (neutrophil collagenase) and MPO, the neutrophil serine protease inhibitor SERPINB1, and multiple components of the membrane/vesicle trafficking machinery (SNAP23, STX8, SNAP29) among the 50 most differentially expressed proteins in cluster 5 suggests persistent neutrophil activation, degranulation, and possibly generation of neutrophil extracellular traps (NETosis). The presence of SAMD9L and MNDA among the top differentially expressed proteins highlights the prominence of the type-I interferon signature noted above and may be indicative of a role for sustained type I IFNs in driving persistent neutrophil activation^35^.

To summarize, broad-based serum proteomic screening identifies a subset of PASC individuals that have evidence of persistent inflammation that is sustained over the time course of this study. The signatures group inflammatory PASC into two major clusters (identified here as 4 & 5). One group (cluster 4) has prominent IFN-γ and evidence of both IFN-γ and NF-kB driven inflammation with upregulation of several inflammation-driven cytokines, chemokines, receptors, and immune checkpoint proteins. The second group (cluster 5) has evidence of ongoing IFN-γ and NF-κB driven inflammation as well but with a more prominent signature of neutrophil activation, degranulation, and/or NETosis paired with evidence for persistent type I IFN signaling. It is unclear whether these represent distinct inflammatory states or are part of a continuum of inflammation and resolution. To address this, we evaluated whether participants who were initially in one cluster at the earliest post-60 day PSO timepoint, transitioned to a different cluster at later timepoints, suggesting a transition to a different inflammatory or non-inflammatory state. We found that most individuals remain in the same cluster over the time course of observation in this study. There were a small number that transitioned within the inflammatory clusters or from inflammatory to non-inflammatory but there was no clear pattern (Fig. S3B). Longer follow-up and a larger cohort may be needed to understand clearly how the different immune states are related in PASC.

### The serum protein signature identified in inflammatory PASC can be validated in an independent cohort

To determine whether these observations could be extended to an independent cohort of PASC participants collected across a broader range of acute COVID severities, we used the INCOV cohort, the only other recent published dataset using Olink technology to evaluate over 442 proteins in blood samples from PASC^36,37^.

The INCOV cohort includes data from 204 SARS-CoV-2-infected participants and 289 healthy controls. Of the 204 INCOV participants, 75 met the criteria used for our cohort (Olink data available from sample obtained ≥60 days after acute infection + clinical data available). Forty-three percent (43%) of these had 1 or more PASC symptoms like the PASC participants in our cohort, and the remainder had no recorded PASC symptoms, similar to the “recovered” group in our cohort. The Olink panel employed in the INCOV study measured only 442 of the 1463 proteins measured in our study but 163 proteins overlapped with the inflammatory signatures that significantly defined the two inflammatory clusters (4 & 5) in our cohort (Supplemental Fig. S11). There are two other differences worth noting: First, in our study, the Olink assay was performed on serum while the INCOV cohort used plasma. Second, while both studies utilized the Olink proximity extension assay (PEA)® technology to detect the serum proteins, this was quantified using a next-gen sequencing approach in our study whereas it was quantified by multiplex quantitative polymerase chain reaction (qPCR) in the INCOV study.

**Fig. 5:**
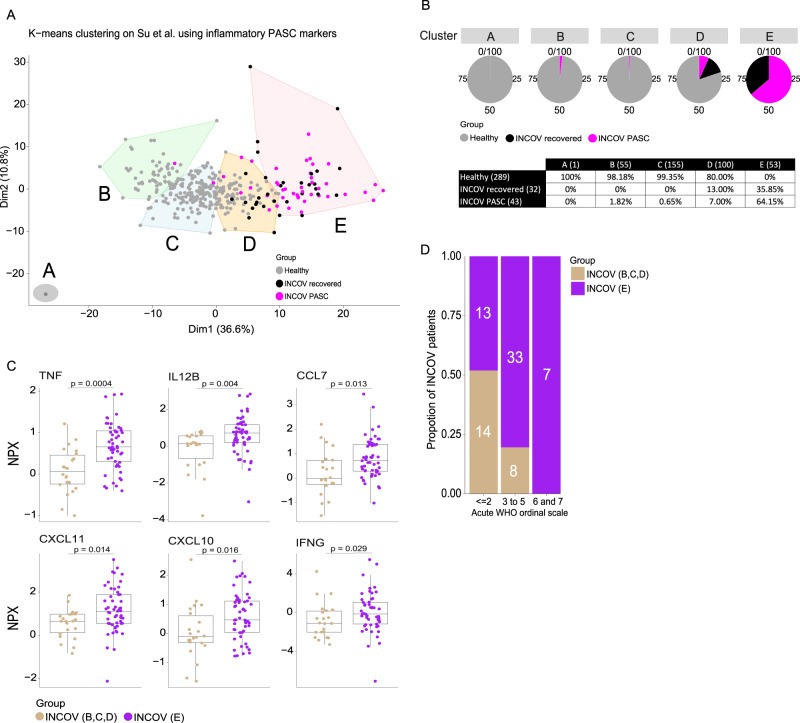
Independent cohort validation of inflammatory PASC signatures. **A** K-means unsupervised clustering of Olink proteomic data from Su Y et al. (2022) showing 5 clusters, **A**–**E** of INCOV participants that consisted of PASC (symptomatic) and recovered (participants showing no PASC symptoms) along with healthy controls. **B** Pie chart representation and table showing the percentage of PASC, recovered and healthy participants within each cluster. The number of participants per cluster (columns) and per group (rows) are represented within brackets. **C** Box and jitter plots of cytokines/chemokines significantly upregulated in the INCOV participants of cluster E (*n* = 53) vs. INCOV participants of clusters **B**, **C**, and **D** (*n* = 22). *P*-values were determined by a two-sided Wilcoxon test. Box plots show the median (centerline), the first and third quartiles (the lower and upper bound of the box) and the whiskers show the 1.5x interquartile range of the data. **D** Distribution of different disease severities, as judged by World Health Organization (WHO) ordinal scale across INCOV participants in cluster E vs INCOV participants in clusters **B**, **C**, and **D**. Y-axis and the numbers in bar graphs represent proportion and number of participants per INCOV group in each WHO scale bin respectively.

Similar to our cohort, k-means unsupervised clustering of the Olink proteomic data from the first time point ≥60 days PSO per INCOV participant (*n* = 75) was performed with k = 5 using the 163 overlapping proteins (Fig. 5A). Like our inflammatory clusters 4 and 5 that consisted primarily of PASC participants (which was 65% of all PASC), 64.2% of the participants in INCOV cluster E were PASC (Fig. 5A and Fig. 5B). No healthy controls clustered with cluster E. INCOV cluster D was made up of a mixture of PASC, recovered, and healthy controls, like our cluster 2. The remaining clusters (A, B, C) were made up predominantly of healthy individuals. INCOV cluster E showed significant enrichment of 129 of the 163 proteins (79%) that defined our inflammatory PASC clusters 4 and 5. Despite the different matrices (serum vs. plasma) and different quantification methods (NGS vs. qPCR) used for the two cohorts, results were robust with cluster E demonstrating elevation of cytokines and chemokines observed in our inflammatory PASC cluster 4 (IFN-γ, IL12, CXCL10, CXCL11, TNF, CCL7, etc.) (Fig. 5C) combined with those elevated in our inflammatory PASC cluster 5 (DDX58, LAMP3, etc.) (Supplemental Fig. S12 & Data S9). The broader diversity of disease severity in the INCOV cohort compared to our mild to moderate cohort, allowed us to make an association between the clinical measure of acute disease severity (WHO ordinal scale score) and proteomic inflammatory signatures. Interestingly, INCOV participants from cluster E predominantly exhibited an acute WHO ordinal score of ≥3 reflecting the association between more severe acute disease and persistent inflammation^37^ (Fig. 5D).

### A predicted panel of 3 proteins exhibits performance characteristics that has potential clinical utility

**Fig. 6:**
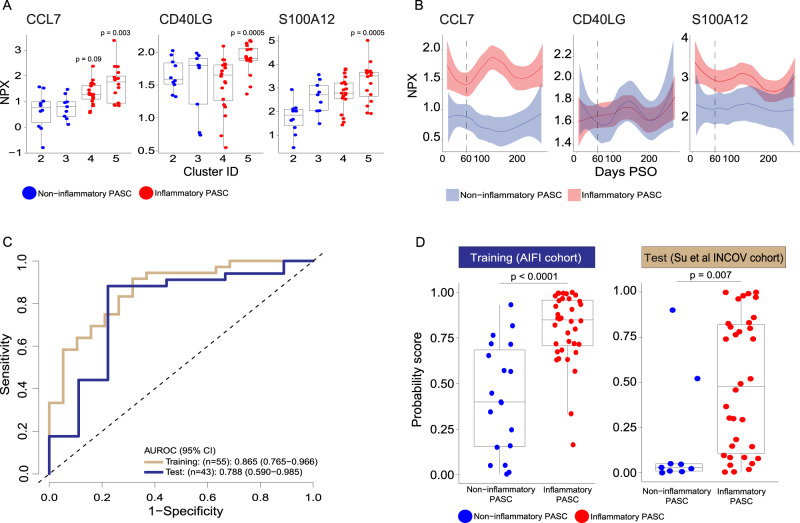
Diagnostic panel for inflammatory vs non-inflammatory PASC. **A** Box and jitter plots of CCL7, CD40LG and S100A12 Normalized Protein Expression (NPX) (y-axis) between PASC in inflammatory clusters 4 and 5 (*n* = 36) and PASC in non-inflammatory clusters 2,3 and 4 (*n* = 19) (x-axis). *P*-values determined by a two-sided Wilcoxon test were calculated comparing inflammatory cluster 4 and inflammatory cluster 5 independently to clusters 2 and 3. **B** Longitudinal Loess fit plots of CCL7, CD40LG and S100A12 NPX on samples available from early acute infection through >250 days post symptom onset (PSO) (x-axis). PASC participants from inflammatory clusters 4 and 5 are represented here as inflammatory PASC (red), PASC participants from clusters 2 and 3 are represented here as non-inflammatory PASC (blue). The bands in the loess smooth fit plots display the 95% confidence interval. **C** Receiver operating characteristic (ROC) curves of a logistic regression (LogReg) model of three proteins (CCL7, CD40L, S100A12) for distinguishing inflammatory versus non-inflammatory PASC and the corresponding areas under the ROC curve (AUROCs) on the training data (*n* = 36 inflammatory and *n* = 19 non-inflammatory) and the test data (INCOV: *n* = 34 inflammatory and *n* = 9 non-inflammatory). **D** Boxplots of the LogReg probability scores distinguishing inflammatory (*n* = 36) versus non-inflammatory (*n* = 19) PASC, left panel in the training dataset and inflammatory (*n* = 34) versus non-inflammatory (*n* = 9) INCOV test data, right panel. *P*-values were determined by a two-sided Wilcoxon test. All box plots show the median (centerline), the first and third quartiles (the lower and upper bound of the box) and the whiskers show the 1.5x interquartile range of the data.

The full protein panel identified in PASC participants with persistent inflammation would be impractical to deploy as a clinical test to differentiate inflammatory and non-inflammatory PASC participants. To design a smaller panel with potential clinical utility, we identified the 35 most significantly differentially expressed proteins distinguishing inflammatory from non-inflammatory PASC in our cohort that were also present and significantly differentially expressed in the INCOV cohort at the first available timepoint ≥60 days PSO. Among the 35 proteins, a list of 15 proteins (Supplemental Data S10) were selected as candidates for a protein panel to distinguish inflammatory versus non-inflammatory PASC. All these proteins measured in both our study and INCOV, were significant in distinguishing the two PASC groups (adjusted *p* < 0.008) in our study and had strong support in the literature as biomarkers for COVID-19 and/or PASC. We tested various combinations of these 15 analytes in a logistic regression (LogReg) model using our PASC cohort as the training dataset (*n* = 36 inflammatory and *n* = 19 non-inflammatory) and the INCOV PASC cohort as the test dataset (*n* = 34 inflammatory and *n* = 9 non-inflammatory) using the first time point available ≥60 days PSO. We identified a three-protein panel (CCL7, CD40LG and S100A12) that performed well to distinguish inflammatory versus non-inflammatory PASC in this model (See Methods & Supplemental Data S10). These proteins were up regulated in the inflammatory clusters at ≥60 days PSO and persisted over time in the inflammatory PASC group compared to the non-inflammatory PASC group, with the exception of CD40LG that was significantly higher only around the 60 days PSO time point, specifically in the inflammatory cluster 5 participants (Fig. 6A and Fig. 6B). The proposed clinical panel of 3 analytes had an area under the receiver operating characteristic curve (AUROC) of 0.865 (95% confidence interval (CI): 0.765–0.966) on the training data and 0.788 (95% CI: 0.590–0.985) on the test data (Fig. 6C). The LogReg probability scores of inflammatory PASC were significantly higher than those of non-inflammatory PASC in both the training (*p* < 0.0001) and the test (*p* = 0.007) datasets (Fig. 6D). A higher LogReg score indicates a higher probability that the participant has inflammatory PASC. Similar results were observed when comparing LogReg scores of inflammatory PASC with those of uninfected and recovered participants (Supplemental Fig. S13A). The LogReg scores of the inflammatory PASC compared to the non-inflammatory PASC remained significantly higher when compared within each days PSO window (Supplemental Fig. S13B). It is encouraging that the potential diagnostic value of this signature holds up in an independent, orthogonal dataset that was measured using a different matrix (plasma vs. serum). The inclusion of a wider range of acute WHO clinical severities by evaluating performance in the INCOV independent test cohort, suggests that this 3-marker diagnostic panel could not only be applied to PASC participants that had mild acute COVID-19 symptoms but also PASC participants that had a more severe acute clinical course. Moreover, a combination of multiple biomarkers that together, are associated with disease may improve both the sensitivity and specificity of a diagnostic test compared to a single biomarker. Although the model suggests that this panel may have clinical utility to identify those PASC participants that have ongoing inflammation, clear choices were made in selection of these biomarkers that may bias their performance in the clinical setting. Additional studies in a larger cohort, over a broader range of timepoints would be needed to validate any panel before it could be used more broadly. We believe that this, or a similar panel, could be used to stratify those that may benefit most from immunomodulatory therapy.

## Discussion

We have identified a serum proteomic signature using a broad-based screen that identifies individuals with PASC that have signs of persistent inflammatory disease. In our cohort, approximately 60% of PASC exhibited an inflammatory signature. Those with evidence of persistent inflammation had a broad range of clinical features that did not clearly segregate the group, suggesting the importance of overlaying biological and clinical readouts in this diverse condition. Our findings provide insights to potential molecular mechanisms of persistent inflammation in PASC and suggest possible therapeutic targets that may be efficacious including JAK inhibitors or specific cytokine blockade in individuals that have the persistent inflammatory protein signature (TNF, IL-6, IFN-γ, etc). Our findings extend previous observations that have variably reported increased expression of IFN-γ, IFN-β, IFN-λ1/2/3, TNF, IL-6, IL-1β, and PTX3 in plasma from PASC participants using targeted cytokine panels^18, 38,39^. While these previous studies grouped all PASC participants, we provide the first evidence that more than half of all PASC have an inflammatory protein signature while others do not have this signature. We show that in inflammatory PASC, the IL-12/IFN-γ axis is highly active and is combined with a NF-κB driven protein signature, possibly activated by TNF, and leading to excess IL-6 expression.

Furthermore, we show evidence of a persistent type I IFN driven protein signature present in the inflammatory PASC proteomic cluster 5 that trends toward normal, approximately 6 months post-infection, paralleling recent reports of persistent SARS-CoV-2 RNA and/or spike protein being detected in serum or non-pulmonary tissues up to 6–12 months after infection^30–32^. Whether persistence of viral products is the driver for ongoing inflammation in PASC remains to be proven but the concept is particularly intriguing. Several papers have highlighted a role for type I IFNs early in disease and the severe clinical outcomes that occur in participants with signaling defects or neutralizing antibodies that target type I IFNs or IFN signaling pathways^40–42^. Under normal circumstances, this is accompanied by transient expression of Type II IFN (IFN-γ) that assists in the generation of adaptive immune responses. However, sustained expression of IFN-γ by cytotoxic T cells or Th1 cells typically requires that these cells recognize antigenic peptides, presented via MHC to their T cell receptors. NK cells or tissue-resident innate lymphoid cells may also express IFN-γ in response to residual viral components. Persistent presence of viral products could serve as the driver of this process leading to a persistent inflammatory protein signature in blood. Links between persistent interferon stimulation and activation of neutrophils is well established in numerous inflammatory diseases and may explain the strong signature of neutrophil degranulation and NETosis we observed in some individuals with inflammatory PASC.

Finally, we show that the serum protein signature we identified can be applied to another independent, orthogonal COVID dataset with samples collected from participants with a range of acute clinical COVID severities to identify PASC participants with persistent inflammatory disease. We have used these data to propose a serum diagnostic panel of three marker proteins (CCL7, CD40LG, S100A12) and have proposed that with further validation, these proteins may be helpful to differentiate inflammatory PASC from non-inflammatory PASC. This could allow participants to be stratified for clinical trials or for immunomodulatory therapies.

## Methods

### Regulatory approvals

Informed consent was obtained from all participants in the Seattle COVID-19 Cohort Study to Evaluate Immune Responses in Persons at Risk and with SARSCoV-2 Infection and to publish individual’s indirect identifiers such as exact age, sex, and BMI. The Fred Hutch Cancer Center Institutional Review Board approved this study (IR10440).

### Study conduct

Serum was collected from participants enrolled in the longitudinal study, “Seattle COVID-19 Cohort Study to Evaluate Immune Responses in Persons at Risk and with SARS-CoV-2 Infection”^43^. Eligibility criteria included adults in the greater Seattle area at risk for SARS-CoV2 infection or those diagnosed with SARS-CoV-2 by a commercially available PCR assay. Study data were collected and managed using REDCap electronic data capture tools hosted at Fred Hutch Cancer Center, including detailed information on symptoms during acute infection and longitudinal follow-up ranging from 33–379 days post symptom onset. All but 2 persons in the “uninfected” group had at least 1 symptom of SARS-CoV-2 infection within the 14 days prior to study screening but had a negative SARS-CoV-2 nasopharyngeal PCR test. Sex of participants was determined based on self-reporting. Participants were not compensated for being in this study.

### Definition of PASC

At the time of study design, the definition of PASC was evolving including some literature that utilized a persistence of symptoms lasting as few as 30 days from symptom onset to define Long COVID. However, we noticed that for many participants, the loss of smell and taste were the sole persistent symptoms in individuals that had otherwise recovered symptomatically after their acute SARS-CoV-2 infection. In most cases, this is resolved by 60 days post symptom onset. To prevent any confounding from acute recovery, we, therefore, included study participants whose symptoms had lasted for at least 60 days from symptom onset. To assure that our cohort was also representative of current definitions of PASC, we determined whether or not they also met the recent WHO Delphi Consensus criteria (at least 60 days of symptoms persisting for a minimum of 90 days post-symptom onset) (https://www.who.int/publications/i/item/WHO-2019-nCoV-Post_COVID-19_condition-Clinical_case_definition-2021.1). We confirmed that all but 1 PASC participant in our cohort continued to experience symptoms for more than 90 days post-symptom onset. The individual without 90-day follow-up left the study and could not be contacted.

### PASC symptom category clustering

We collected symptom information from each donor over multiple visits. Participants reported symptoms ranging from fatigue and fever to more clinically concerning symptoms like arrhythmia and brain fog. Each individual PASC symptom that was reported at ≥60 days PSO were merged into six major categories including fatigue/malaise, pulmonary, cardiovascular, gastrointestinal, musculoskeletal, and neurologic. Other mild symptoms were combined into a single category as “any mild symptoms” (Supplemental Data S1). We acknowledge that these symptoms are reported and collected in snapshots of time and may not fully capture the day-to-day variability.

The more clinically concerning symptoms were combined into organ-related symptom groups as follows:

Fever/Chills = Fever 101 F or greater and/or Chills

Fatigue/Malaise = Fatigue or malaise

Pulmonary = Dry cough and/or SOB or DIB and/or Chest congestion and/or Chest tightness and/or Oxygen supplementation

Cardiovascular = Arrhythmias and/or Palpitations but not Tachycardia

Gastrointestinal = Diarrhea and/or Nausea or vomiting

Musculoskeletal = Muscle aches or pains and/or arthralgias and/or arthritis

Neurologic = Brain fog and/or Memory loss

Tinnitus = Tinnitus

All other non-specific symptoms were combined into the “any mild symptom” category, which include the following:

Sore throat

Scratchy throat

Low fever under 101 F

Runny nose

Loss of smell

Loss or change in taste

Headache

Itchy eyes

Dry lips in corners (angular cheilitis)

Tachycardia

Dizziness

Lightheadedness

Hair loss/Alopecia

Hands or feet tingling

Diaphoresis or sweating

Symptom information was converted to binary format where yes = 1 and no = 0. Missing symptom information is denoted by NA. For each symptom category we identified symptom-specific differential serum proteins using a linear mixed model utilizing all longitudinal timepoints available for each participant post ≥60 days PSO. We used the lme4 package (v1.1) to carry out linear mixed model analysis where age and sex were fixed variables and donor information was a random variable^44^.

1 $${{{{{\rm{NPX}}}}}} \sim {{{{{\rm{Symptom}}}}}}\,{{{{{\rm{status}}}}}}+{{{{{\rm{Age}}}}}}+{{{{{\rm{Sex}}}}}}\,+(1|{{{{{\rm{Donor}}}}}})$$

The *p*-value is obtained from chi-square statistics. The specific symptom category associated with differential plasma proteins selected using *p* < 0.05. The identified differential proteins from six symptom specific categories were merged together and their expression visualized in a heatmap using package ComplexHeatmap (v2.4).

### Symptom activity metrics and scoring for mild to moderate acute COVID symptoms

Symptom activity in mild to moderate acute COVID was classified by participant report of impact on Activities of Daily Living (ADLs) for each day of illness. Days hospitalized were recorded as were any treatment or therapies received. Participants were scored according to their maximum symptom activity for each day: 0, no symptoms; 1, mild impact on ADLs reported; 2, moderate impact on ADLs reported; 3, severe illness without hospitalization; 4, severe illness with hospitalization; 5, hospitalized with ICU care, or 6, life threatening illness. Duration was assigned for days spent at each level of symptom activity. A clinical activity score was calculated for each participant by multiplying the symptom activity score by the number of days spent at each level, then summing all values.

### Sample processing

Blood was drawn into a serum separator tube and serum samples were processed, aliquoted and frozen within 4 h of blood draw.

### Olink serum protein measurement

Serum samples were inactivated with 1% Triton X-100 for 2 h at room temperature according to the Olink COVID-19 inactivation protocol. Inactivated samples were then run on the Olink Explore 1536 platform, which uses paired antibody proximity extension assays (PEA) and a next generation sequencing (NGS) readout to measure the relative expression of 1472 protein analytes per sample. Analytes from the inflammation, oncology, cardiometabolic, and neurology panels were measured. For plate setup, samples were randomized across plates to achieve a balanced distribution of age and gender. Longitudinal samples from the same participant were run on the same plate. To ensure consistent results between batches, 42 previously run serum samples from the initial batch were included on the second Olink batch as bridging controls. Per protocol, samples were run alongside a negative control (buffer), plate control (pooled serum), and a sample control (pooled serum).

Olink’s standard data normalization was performed on this dataset. Protein expression values were first normalized across wells using an internal extension control (IgG antibodies conjugated with a matching oligo pair). Plates were then standardized by normalizing to the inter-plate pooled serum controls run in triplicate on each plate. Data were then intensity normalized across all cohort samples. Final normalized relative protein quantities were reported as log2 normalized protein expression (NPX) values.

### Olink data preprocessing

Olink results and QC flags were reviewed for overall quality. Internal controls added at the incubation (non-human antigen+antibody pair) and detection (double-stranded DNA) steps were used to ensure consistency in the assay procedure among samples. There were no sample or assay failures flagged in the dataset. Warned samples were observed on some marker subsets (internal controls differ +/- 0.3 NPX from plate sample median) but were retained in the analysis. Results for TNF, IL-6 and CXCL8, which were measured on all 4 Olink panels, were reviewed prior to averaging to a single NPX value for analysis. Two samples had discrepant cross-panel measurements on these proteins. The results that trended most consistently with the participant’s longitudinal measurements were kept and averaged. Serum samples were analyzed in two batches. Following the method recommended by Olink, results of the later batch were bridged to those of the earlier batch using a set of 42 cohort samples that were tested in both batches. A batch offset for each analyte was calculated as the median batch-to-batch difference on the 42 serum samples, excluding samples with QC warning flags. The analyte-specific offsets were then added to the raw NPX values of the later batch.

### Antibody ELISAs for RBD

Half-well area plates (Greiner) were coated with purified RBD protein at 16.25 ng/well (kind gift from Dr.Leo Stamatatos^45^) in PBS (Gibco) for 14–24 hr at room temperature. After 4 x 150 ul washes with 1X PBS, 0.02% Tween-20 (Sigma) using the BioTek ELx405 plate washer, the IgA and IgG plates were blocked at 37 °C for 1-2 h with 1X PBS, 10% non-fat milk (Lab Scientific), 0.02% Tween-20 (Sigma); IgM plates were blocked with 1X PBS, 10% non-fat milk, 0.05% Tween-20. Serum samples were heat inactivated by incubating at 56 °C for 30 minutes, then centrifuged at 10,000 x *g* for 5 min, and stored at 4 °C before use in the assay. For IgG ELISAs, serum was diluted into a blocking buffer in 7–12 1:4 serial dilutions starting at 1:50. For IgM and IgA ELISAs, serum was diluted into 7 1:4 serial dilutions starting at 1:12.5 to account for their lower concentration. A qualified pre-pandemic sample (negative control) and a standardized mix of seropositive sera (positive control) were run in each plate and used to define passing criteria for each plate. All controls and test sera at multiple dilutions were plated in duplicate and incubated at 37 °C for 1 h, followed by 4 washes in the automated washer. 8 wells in each plate did not receive any serum and served as blocking controls. Plates then were plated with secondary antibodies (all from Jackson ImmunoResearch) diluted in blocking buffer for 1 h at 37 °C. IgG plates used donkey anti-human IgG HRP diluted at 1:7500 (cat # 109-035-098); IgM plates used goat anti-human IgM HRP diluted at 1:10,000 (cat # 109-035-043); IgA plates used goat anti-human IgA HRP at 1:5000 (cat # 109-035-011). After 4 washes, plates were developed with 25 ul of SureBlock Reserve TMB Microwell Peroxide Substrate (Seracare) for 4 min, and the reaction stopped by the addition of 50 ml 1 N sulfuric acid (Fisher) to all wells. Plates were read at OD_450nm_ on a SpectraMax i3X ELISA plate reader within 20 min of adding the stop solution.

OD_450nm_ measurements for each dilution of each sample were used to extrapolate RBD endpoint titers when CVs were less than 20%. Using Excel, endpoint titers were determined by calculating the point in the curve at which the dilution of the sample surpassed that of 5 times the average OD_450nm_ of blocking controls + 1 standard deviation of blocking controls.

RBD titers at day 60 PSO were estimated by a linear mixed effects model of titers over time from day 30 PSO with random effects for the intercept and slope, using lme from the nlme R package.

### Intracellular cytokine staining (ICS) assay

Flow cytometry was used to examine SARS-CoV-2-specific CD4+ and CD8 + T-cell responses using a validated ICS assay. The assay was similar to a published report^43,46,47^. Peptide pools covering the structural proteins of SARS-CoV-2 were used for the six-hour stimulation. Peptides matching the SARS-CoV-2 spike sequence (316 peptides, plus 4 peptides covering the G614 variant) were synthesized as 15 amino acids long with 11 amino acid overlaps and pooled in 2 pools (S1 and S2) for testing (BioSynthesis). All other peptides were 13 amino acids long, overlapping by 11 amino acids and were synthesized by GenScript. The peptides covering the envelope (E), membrane (M) and nucleocapsid (N) were initially combined into one peptide pool, but most of the assays were performed using a separate pool for N and one that combined only E and M. Several of the open reading frame (ORF) peptides were combined into two pools, ORF 3a and 6, and ORF 7a, 7b and 8. All peptide pools were used at a final concentration of 1 microgram/ml for each peptide. As a negative control, cells were not stimulated, only the peptide diluent (DMSO) was included. As a positive control, cells were stimulated with a polyclonal stimulant, staphylococcal enterotoxin B (SEB). Cells expressing IFNγ and/or IL-2 and/or CD154 were the primary immunogenicity endpoint for CD4 + T cells and cells expressing IFNγ were the primary immunogenicity endpoint for CD8 + T cells. The overall response to SARS-CoV-2 was defined as the sum of the background-subtracted responses to each of the individual pools. A sample was considered positive for CD4+ or CD8 + T cell responses to SARS-CoV-2 if any of the CD4+ or CD8 + T cell responses to the individual peptide pool stimulations was positive. Positive responses to a given peptide pool stimulation were determined using the MIMOSA (Mixture Models for Single-Cell Assays) method ^48^. The MIMOSA method uses Bayesian hierarchical mixture models that incorporate information on cell count and cell proportion to define a positive response by comparing peptide-stimulated cells and unstimulated negative controls. MIMOSA estimates the probabilities that peptide-stimulated responses are responders and applies a false-discovery rate multiplicity adjustment procedure^49^. Responses with false-discovery rate *q*-values < 0.05 were considered positive. The total number of CD4 + T cells must have exceeded 10,000 and the total number of CD8 + T cells must have exceeded 5000 for the assay data to be included in the analysis (supplemental Data S11 and Fig. S14).

### Identification of pathways with high rule-in performance

Partial area under the receiver operating characteristic curve (pAUC)^50,51^ was used to evaluate the rule-in performance^15^ of individual pathways in identifying PASC participants with respect to recovered and uninfected participants. The pAUC bounded by a specificity between 90–100% and the corresponding 99% confidence interval (two-sided) of each pathway were calculated using the “*ci.auc*” function in the R package *pROC* with the following parameters: partial.auc = c(0.9, 1), conf.level = 0.99, boot.n = 1000. A pathway was identified as significant with *p* < 0.01 if its pAUC lower confidence bound was above the corresponding pAUC of a random, non-performing classifier, i.e. 0.005.

We collected the canonical pathway “c2.cp.v7.2.symbols” genesets collection and associated gene information from MsigDB (v7.2). The 2871 canonical pathways were used to perform single sample GSEA (ssGSEA)^52^ using GSVA (v1.40) R package. Among 2871 pathways, 1960 pathways with overlapping serum proteins were used as input for GSVA with min.size 2 and max.size 2000 genes as parameters. The ssGSEA resulted in a normalized enrichment score (NES) for each pathway. One sample for each PASC donor was selected as follows: The first time point ≥60 days PSO for infected PASC donors (*n* = 55) and the last time point ≥60 days PSO for individuals that were infected but recovered (*n* = 24). Including the uninfected individuals (*n* = 22), a single sample from each of 101 donors was included in the biomarker analysis.

The rule-in approach implemented to identify pathways significantly associated with PASC donors was performed as follows: Parameters including confidence interval (CI), pAUC and bootstrap (boot.n) were used. Bootstrap analysis was performed using random seed over multiple processors using the R function mcapply (v3.4.1). Range of CI 0.8–0.99 and pAUC 0.8–0.95 was used to identify pathways associated with the PASC group. These pathways were used to differentiate the uninfected and PASC donors into separate clusters incorporating >50% of cluster size. The clustering was performed by the k-means approach implemented in ComplexHeatmap (v2.4) and visualized. The bootstrap analysis resulted in CI of 0.99 and pAUC of 0.95 which can differentiate uninfected and PASC donors in clusters. These parameters were used to identify pathways associated with PASC with a bootstrap of 1000 as noted above. The analysis resulted in 85 pathways. These 85 pathways were then collapsed into 54 modules.

A module is defined if pairwise genests had an overlap of at least 25% (jaccard index 0.25) of the genes/proteins between them using the Enrichment Map approach^16^. The 54 modules were then used to perform module enrichment at a single sample level using GSVA. The normalized enrichment score for each module was scaled and clustered using k-means clustering implemented in ComplexHeatmap (v2.4) with parameter row_km and column_km. The identified clusters are then visualized in a heatmap.

### Analysis of Su Y et al. (2022) INCOV Olink data

The INCOV proteomics data was generated using plasma samples and our proteomics data was generated using serum samples. Both used the PEA® technology but were quantified using two different assays, (NGS for our cohort and multiplex qPCR for the INCOV cohort). Olink advises against combining such datasets for analysis, so they were not combined. However, since both platforms measure relative protein abundance, the analysis was performed independently within each cohort to determine whether the same patterns and trends of protein abundance and a similar proteomic signature can be observed across groups. The Olink proteomic data from Su Y et al. consisted of 204 SARS-CoV-2 (INCOV) participants and 289 healthy controls. The INCOV participants were studied at clinical diagnosis (T1), acute disease (acute, T2), and 2–3 months post onset of initial symptoms (convalescent, T3). Olink plasma proteomic data was available for a total of 443 proteins. Among these, 163 proteins overlapped with the differentially expressed proteins found in inflammatory signatures that significantly defined clusters 4 & 5 in our cohort. K-means unsupervised clustering of the INCOV Olink proteomic data was performed on the 163 protein overlap. To remain consistent with our cohort, we used only those samples available for timepoints ≥60 days PSO per INCOV participant (74 INCOV participants met these criteria). If more than one sample was available ≥60 days PSO, we used the sample closest to the ≥60 day timepoint. The k-means function of the stats R package was used with k = 5, allowing 100 iterations.

### Generation of a proposed protein panel to distinguish inflammatory versus non-inflammatory PASC

A list of fifteen proteins (Supplemental Data S10) were selected as candidates for a protein panel to distinguish inflammatory versus non-inflammatory PASC. Several criteria had to be met for inclusion of proteins in this group: All proteins had to be measured in both our study and INCOV, were significant in distinguishing the two PASC groups (adjusted *p* < 0.008) in our study cohort and had strong support in the literature as biomarkers for COVID-19 and/or PASC. Logistic regression (LogReg) models^53^ were used to combine protein measurements into LogReg probability scores as follows:

2 $$W={c}_{0}+\mathop{\sum }\limits_{i=1}^{n}{ci}*{{NPX}}_{i},$$

3 $${score}=\frac{1}{1+{\exp }^{-W}}$$

where ${c}_{0}$ is the intercept, $n$ is the protein number, ${c}_{i}$ is the coefficient of protein $i$, and ${{NPX}}_{i}$ is the NPX value of protein $i$. The models were assessed on our PASC data and evaluated in a stepwise backward elimination procedure: In each step, the approach of 10-fold cross validation was repeated 10,000 times to train LogReg models and evaluate the corresponding p values of individual proteins having a non-zero coefficient. The protein having the highest p-value in the step was eliminated. This procedure was repeated until all proteins had *p* < 0.05. Coefficients of the final LogReg model were then set to the corresponding median coefficients of the remaining proteins.

### Statistics and reproducibility

Challenges associated with sample collection, supply-chain difficulties, and a shifting research environment during the global COVID-19 pandemic required that the Olink analysis be performed in two batches that impacted batch design. The first batch was collected early in the pandemic (April 2020) with the aim of studying the difference between SARS-CoV-2 infected (*n* = 15) versus uninfected donors (*n* = 22). We later found out two of the fifteen infected donors had PASC and had persistent inflammatory signatures over time, which motivated us to expand our study by including a larger cohort of PASC. By the time the second batch was collected for analysis, PASC had become a major concern in COVID-19 patients, so we added an additional 53 PASC donors and 11 recovered COVID-19 donors in the second batch based on sample availability. Based on this, the two batches have a bias related to disease status (PASC, recovered, and uninfected; *p* < 0.05) but no biases on biological sex or age (*p* > 0.05). Longitudinal samples were collected from participants based on their availability, but no replicate samples were collected at any single timepoint. Hence, no statistical method was used to predetermine sample size. We were not blinded to allocation and outcome assessment. To minimize possible batch effects, we used 42 cohort samples as bridging controls for batch correction between the two batches as described in the ‘Olink data preprocessing’ section of the methods. No data were excluded from the analyses.

All statistical analyses were performed using the corresponding functions in RStudio (version 4.1). Comparisons of single protein Olink NPX or module ssGSEA scores between groups were tested using a two-sided Wilcoxon rank sum test and when appropriate, the Benjamini-Hochberg method was applied to adjust *p*-values in multiple-testing correction. Unless specified, an adjusted *p*-value of 0.05 was considered significant and *p*-values less than 0.0001 were reported as *p*-value < 0.0001. All figure panels were assembled using Adobe Illustrator (version 27.2; https://www.adobe.com/products/illustrator.html).

### Reporting summary

Further information on research design is available in the Nature Portfolio Reporting Summary linked to this article.

## Supplementary information

Supplementary Information

Description of Additional Supplementary Files

Supplementary Data 1

Supplementary Data 2

Supplementary Data 3

Supplementary Data 4

Supplementary Data 5

Supplementary Data 6

Supplementary Data 7

Supplementary Data 8

Supplementary Data 9

Supplementary Data 10

Supplementary Data 11

Reporting Summary

## Data Availability

The processed Olink data generated in this study, the processed Su Y et al. (2022) INCOV Olink data, the sample metadata for both studies, the input canonical genesets database, “c2.cp.v7.2.symbols”, from MsigDB (v7.2) and source data files for figures have been deposited in Zenodo^54^ [10.5281/zenodo.7872791]. The processed Olink data generated in this study is also provided in the supplementary data files, supplemental Data S2 and S6. Source data are provided with this paper.

## Source data

Source Data

## Code availability

R Code used to perform analysis and generate figures for this study are made available in Zenodo^54^ [10.5281/zenodo.7872791] and the GitHub repository https://github.com/aifimmunology/PASC-proteomics-talla-vasaikar-et-al.
