# Supplementary material for: Persistent serum protein signatures define an inflammatory subcategory of long COVID
Source: Nat Commun. 2023 Jun 9;14:3417. doi: 10.1038/s41467-023-38682-4 (PMC10252177; doi:10.1038/s41467-023-38682-4)
Supplement: Supplementary file 1 — Supplementary Information [file 41467_2023_38682_MOESM1_ESM.pdf]

Persistent serum protein signatures define an inflammatory subcategory of long COVID

Aarthi Talla[1], Suhas V. Vasaikar[1,2], Gregory Lee Szeto[1,2], Maria P. Lemos[3], Julie L. Czartoski[3], Hugh MacMillan[3], Zoe Moodie[3], Kristen W. Cohen[3,4], Lamar B. Fleming[3], Zachary Thomson[1], Lauren Okada[1], Lynne A. Becker[1], Ernest M. Coffey[1], Stephen C. De Rosa[3], Evan W. Newell[3], Peter J. Skene[1], Xiaojun Li[1], Thomas F. Bumol[1], M. Juliana McElrath[2], Troy R. Torgerson[1]

Affiliations
[1]Allen Institute for Immunology; Seattle, USA
[2]Current Address: Seagen, Bothell, WA
[3]Vaccine and Infectious Disease Division, Fred Hutchinson Cancer Center, Seattle, USA
[4]Current Address: Moderna, Cambridge, MA

Supplementary Information

Supplementary Figure S1

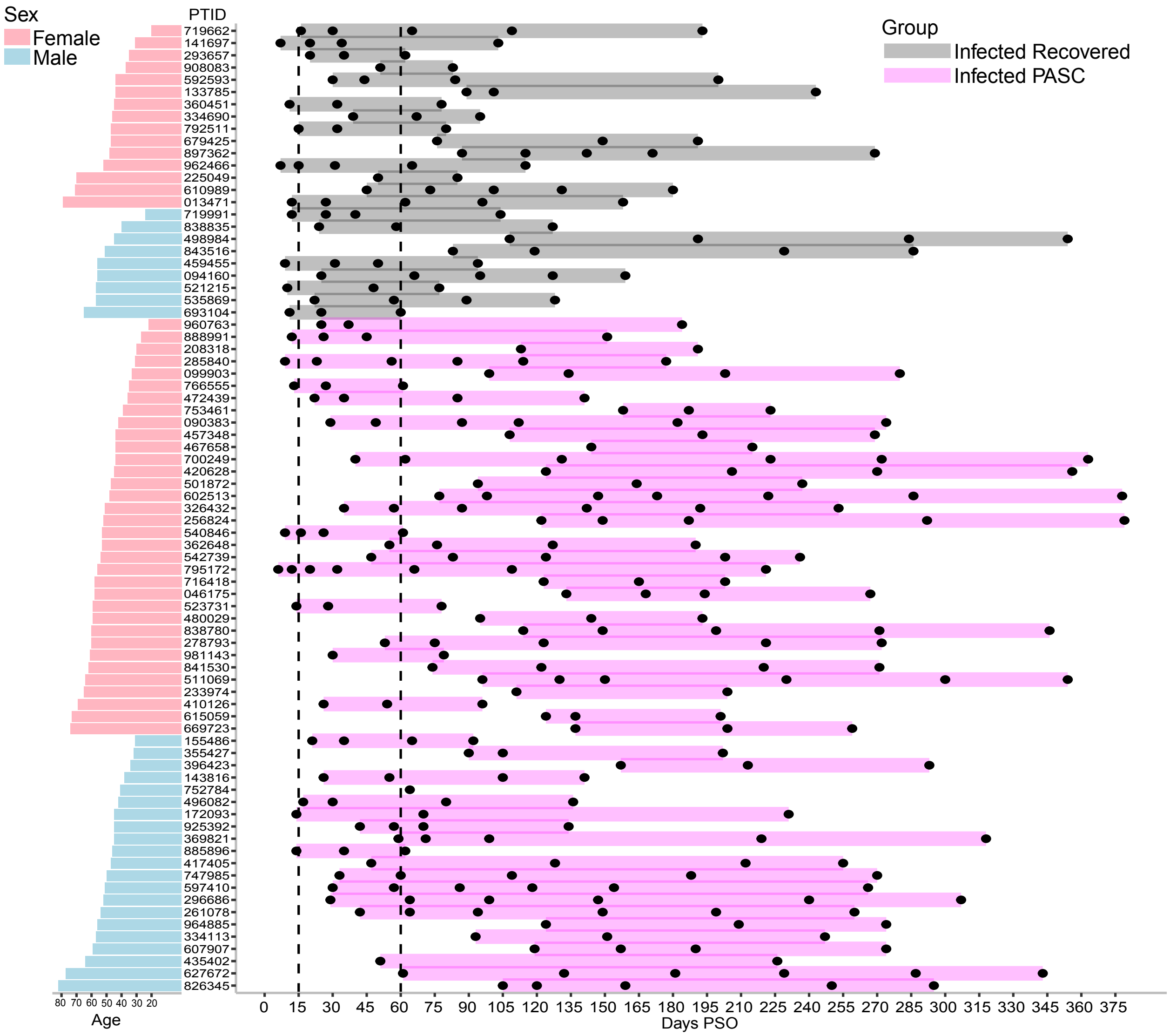

Figure S1. Longitudinal sampling timeline across infected participants. Serum was collected at 2-5 timepoints for each participant ranging from 6 to 379 days PSO. Participants are arranged by sex and increasing age for recovered and PASC.

Supplementary Figure S2

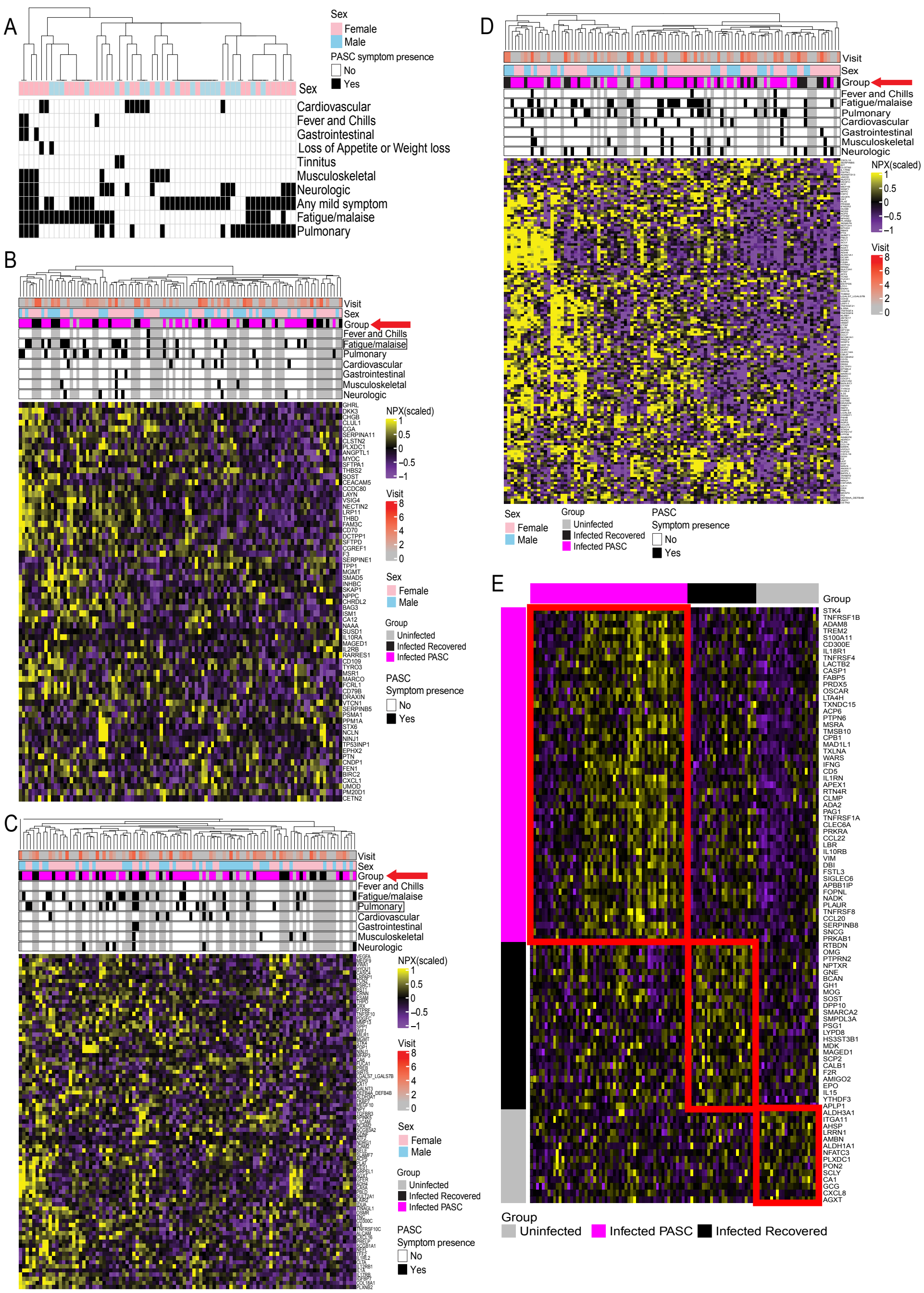

Figure S2. PASC symptomatology alone at convalescence does not clearly drive significant participant clustering. (A) Hierarchical clustering heatmap of symptomatology across infected PASC participants at ≥60 days post symptom onset (PSO). (B,C) Heatmap of the Olink serum proteins significantly associated with Fatigue/Malaise and pulmonary symptoms respectively among infected PASC participants at ≥60 days PSO. The uninfected and infected recovered participants were included to perform hierarchical clustering on these proteins. (D) Heatmap of the union of Olink serum proteins significantly associated with each of the symptoms among infected PASC participants at ≥60 days PSO. The uninfected and infected recovered participants were included to perform hierarchical clustering on these proteins. (E) Heatmap of the top 50 significantly expressed proteins (rows) (p-value < 0.05) in all PASC, recovered (at their first time point available ≥60-days post symptom onset) and uninfected participants. The p-values were determined by comparing expression of a protein in one group to the expression of the same protein in all other groups using a two-sided Wilcoxon test.

Supplementary Figure S3

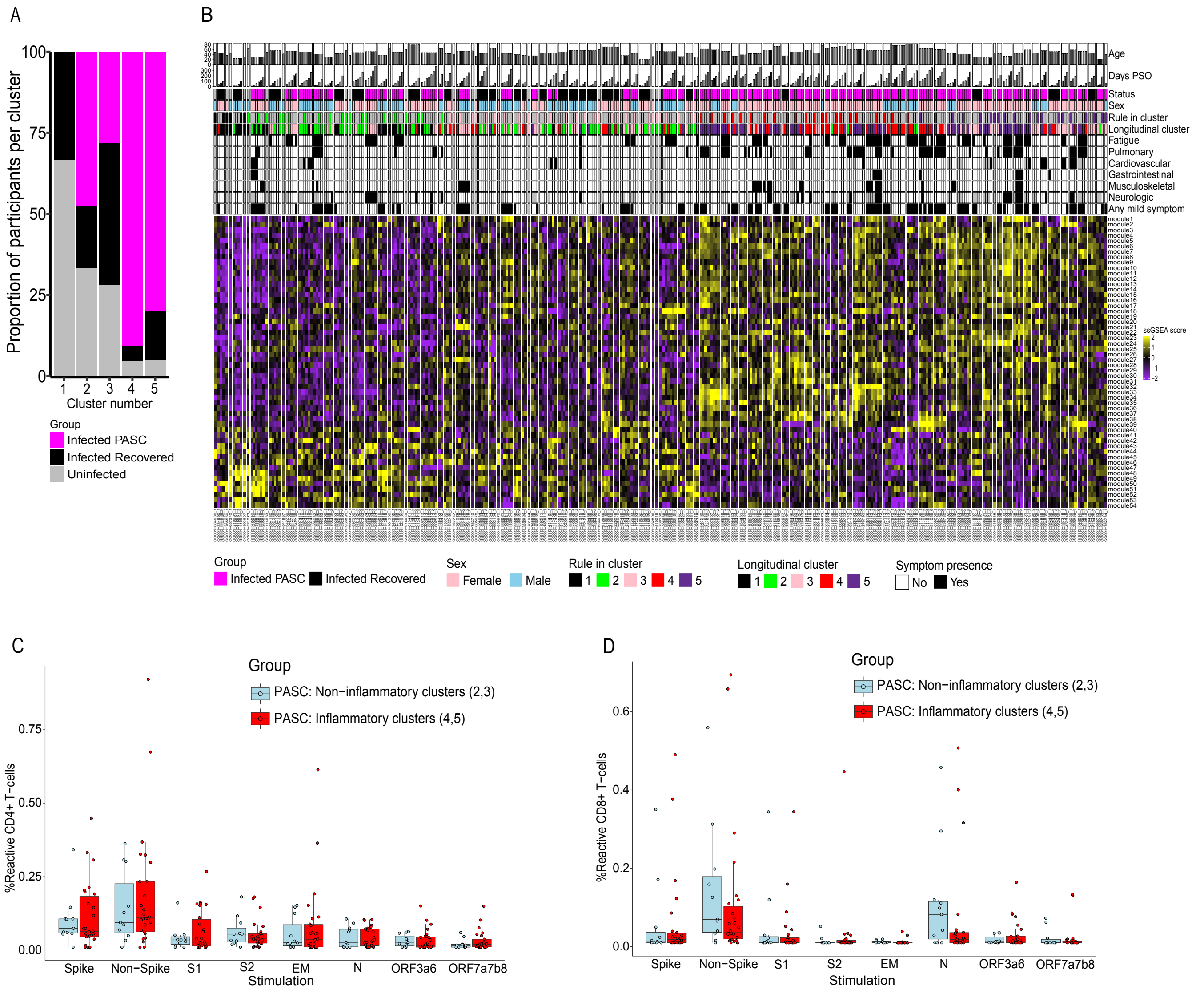

Figure S3. Clustering of the serum proteome identifies inflammatory and non-inflammatory PASC categories. (A) Stacked bar plot of the proportion of uninfected, recovered and PASC participants per cluster identified with the rule-in approach (B) Heatmap of the expression of modules (rows) across the full longitudinal cohort of samples (columns). (C,D) Box plots of SARS-CoV-2 specific CD4+ and CD8+ T-cells between the inflammatory PASC (PASC from clusters 4 and 5, n=36) and non-inflammatory PASC (PASC from clusters 2 and 3, n=19) respectively. Box plots show the median (centerline), the first and third quartiles (the lower and upper bound of the box) and the whiskers show the 1.5x interquartile range of the data.

Supplementary Figure S4

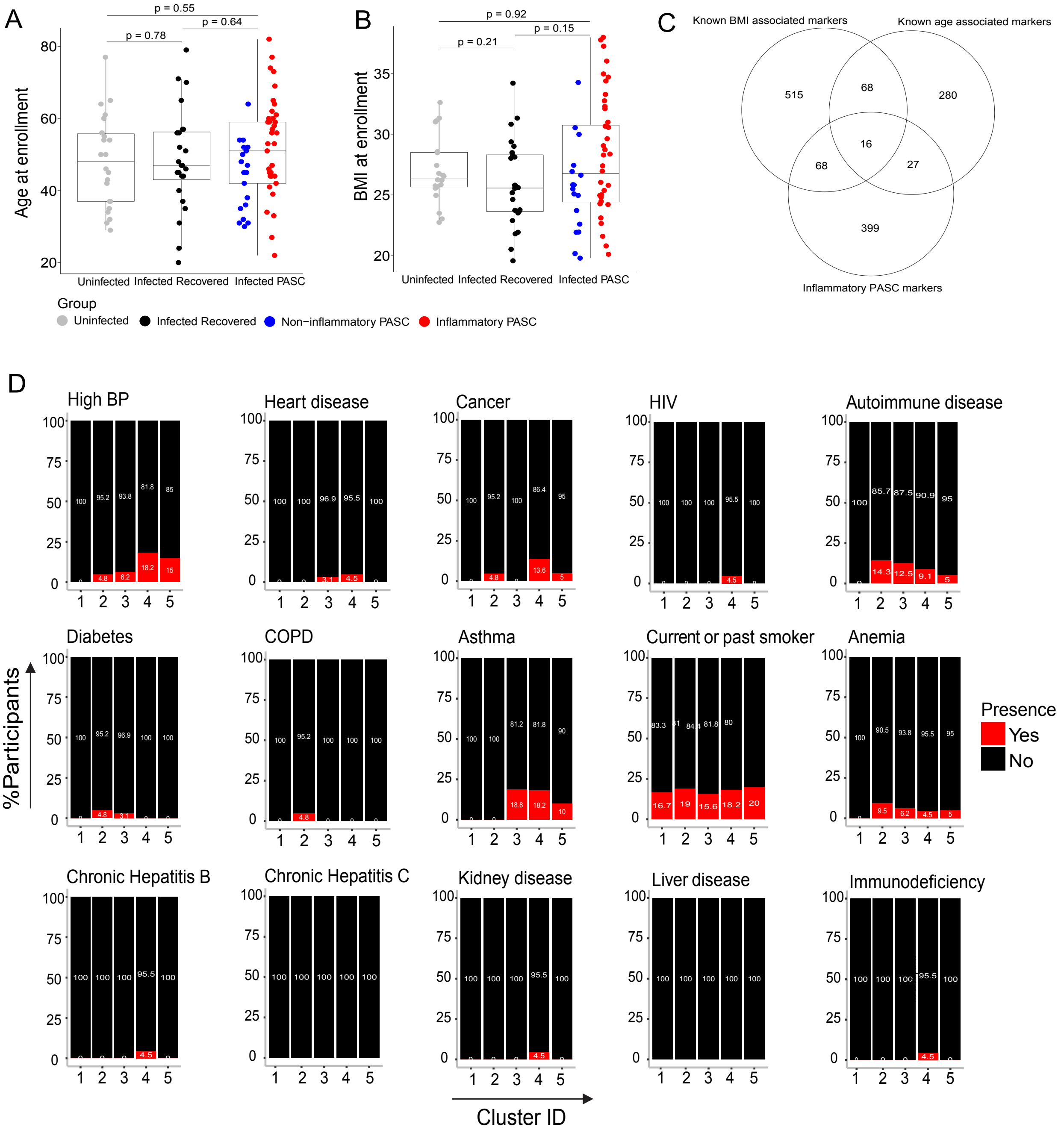

Figure S4. Association of serum proteome clusters with Body Mass Index (BMI) and Age. (A,B) Box and jitter plots of age and BMI at enrollment in the uninfected (n=22), recovered (n=24) and PASC (n=55) participants. The PASC participants are color coded by non-inflammatory and inflammatory PASC. P-values between groups were determined by a two-sided Wilcoxon test. Box plots show the median (centerline), the first and third quartiles (the lower and upper bound of the box) and the whiskers show the 1.5x interquartile range of the data. (C) Venn diagram of the overlapping and unique proteins between the known curated age and BMI associated markers and the inflammatory PASC cluster markers. (D) Stacked bar plots of the proportion of the presence or absence of comorbidities reported during enrollment per cluster of participants.

Supplementary Figure S5

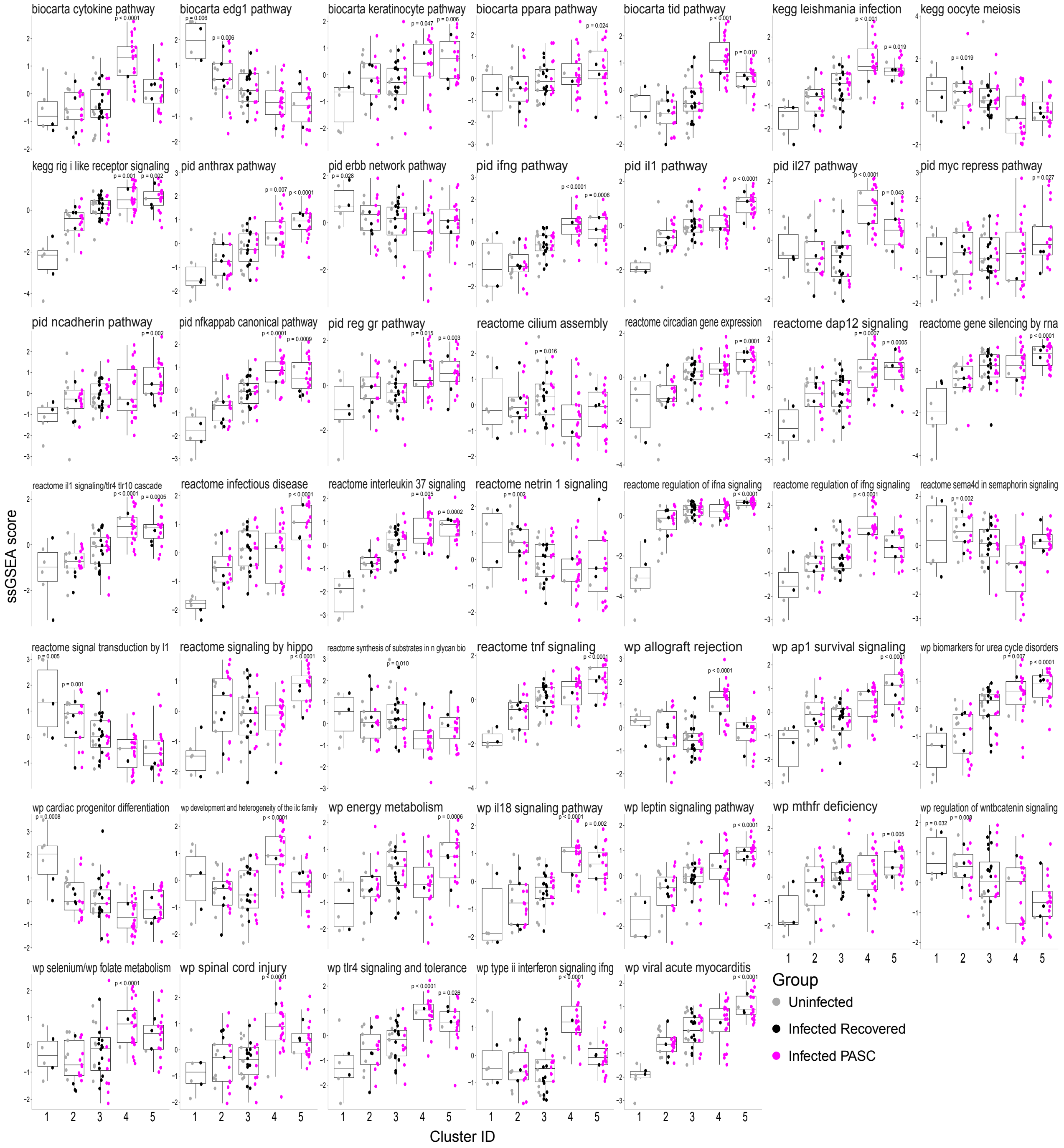

Figure S5. Signaling modules that significantly differentiate the five participant clusters. Box and jitter plots of the Single Sample Gene Set Enrichment Analysis (ssGSEA) scores (y-axis) across all clusters (that consist of PASC, n=55; recovered, n=24; uninfected, n=22 participants) (x-axis) for the modules that were significantly associated with each cluster. P-values were determined by a two-sided Wilcoxon test. All box plots show the median (centerline), the first and third quartiles (the lower and upper bound of the box) and the whiskers show the 1.5x interquartile range of the data.

Supplementary Figure S6

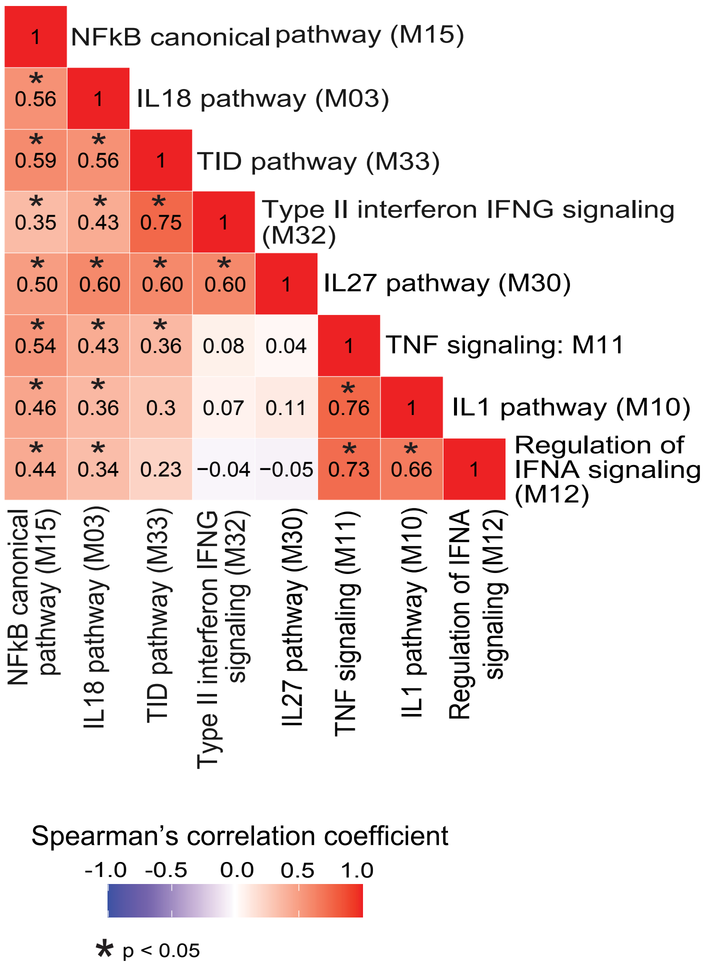

Figure S6. Coordinated activation of immune cascades associated with inflammation in PASC. Pair-wise Spearman's correlation coefficient (two-sided test) heatmap between top enriched modules that define inflammatory clusters 4 and 5 demonstrating co-enrichment of modules.

Supplementary Figure S7

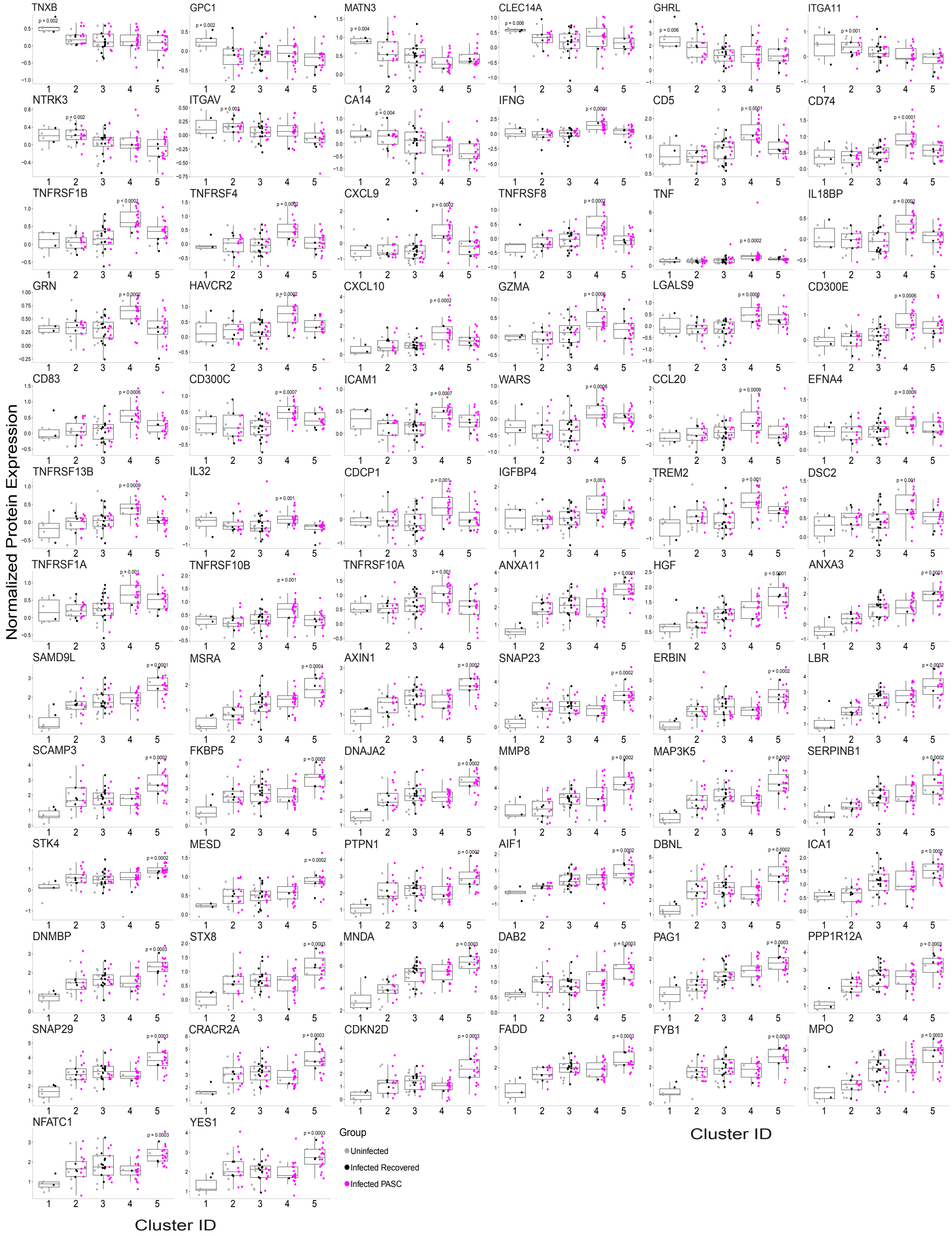

Figure S7. Independent serum proteins that significantly differentiate the five participant clusters. Box plots of the Normalized Protein Expression (NPX) (y-axis) across all clusters (that consist of PASC, n=55; recovered, n=24; uninfected, n=22 participants) (x-axis) of the proteins that were significantly associated with each cluster. P-values were determined by a two-sided Wilcoxon test. All box plots show the median (centerline), the first and third quartiles (the lower and upper bound of the box) and the whiskers show the 1.5x interquartile range of the data.

Supplementary Figure S8

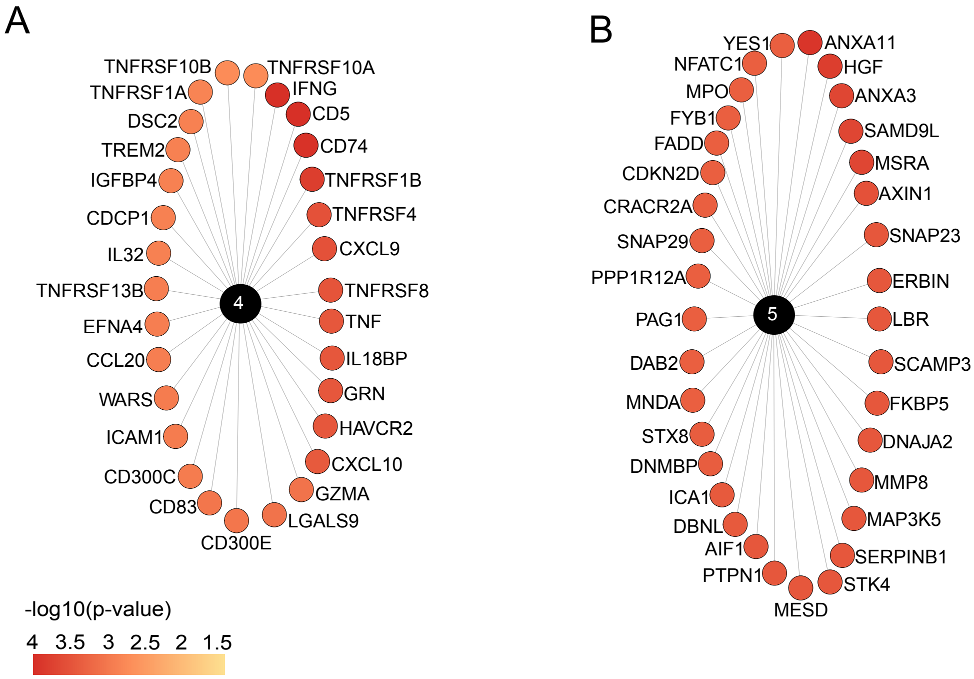

Figure S8. (A) Top 20 proteins significantly up-regulated in cluster 4 and (B) cluster 5 relative to all other clusters. The color gradient of each node represents the -log10 adjusted p-value (<0.05). P-values were determined by a two-sided Wilcoxon test.

Supplementary Figure S9

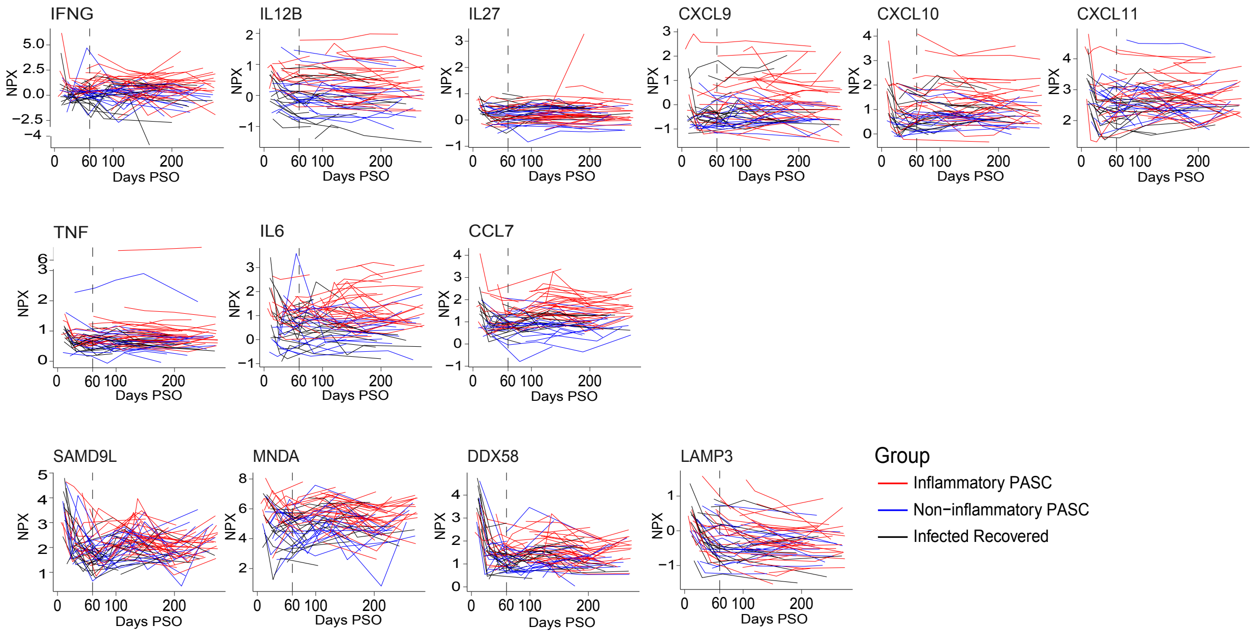

Figure S9. Longitudinal protein expression of interferon and its associated proteins shows persistence over time. Line plots of IFN-γ, TNF, their related cytokines and chemokines and type I interferon associated protein's Normalized Protein Expression (NPX) values (y-axis) on samples available from 6 days post symptom onset (PSO) through >60 days PSO (x-axis). PASC participants from the inflammatory clusters 4 and 5 are represented here as inflammatory PASC (red), PASC participants from clusters 2 and 3 are represented here as non-inflammatory PASC (blue) while the recovered participants are represented in black.

Supplementary Figure S10

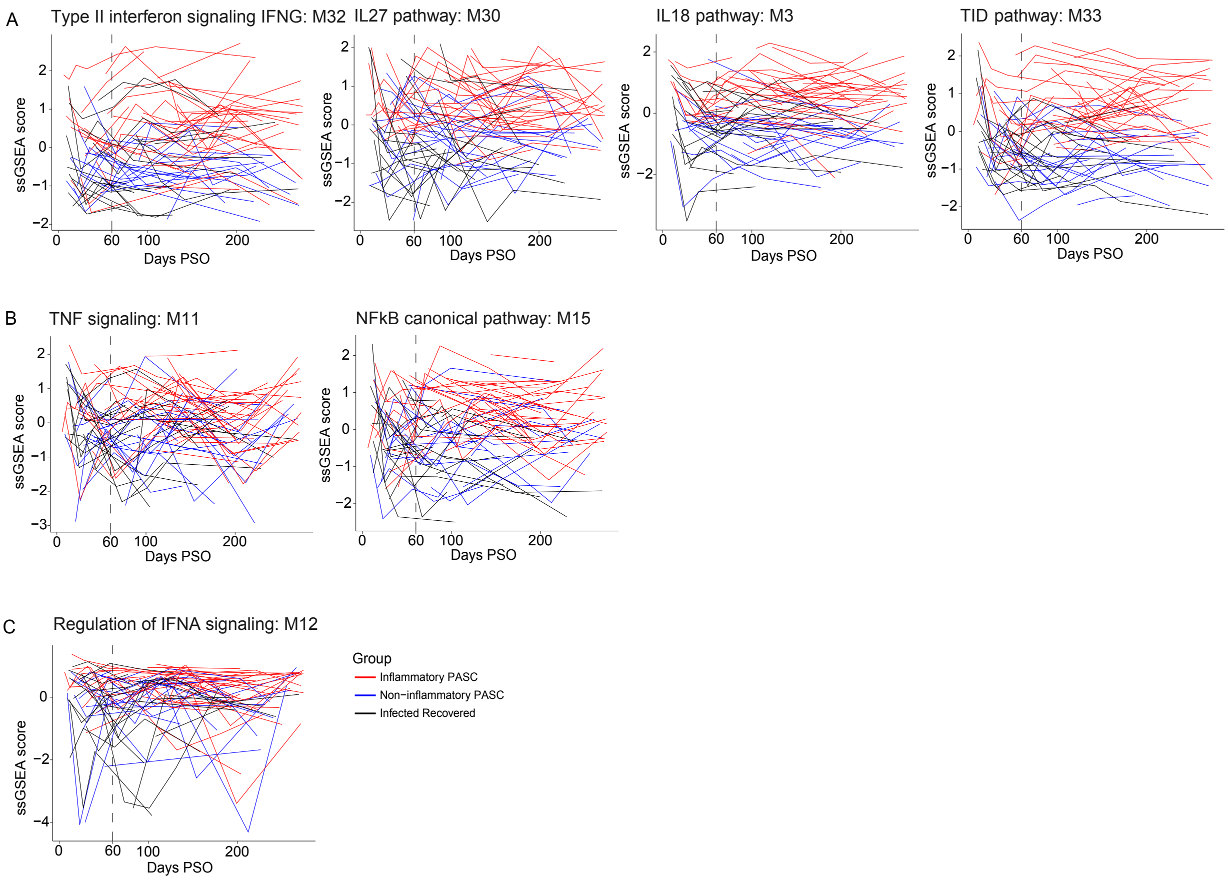

Figure S10. Longitudinal module expression of interferon and its associated signaling modules shows persistence over time. (A,B) Longitudinal module expression (Single Sample Gene Set Enrichment Analysis (ssGSEA) score as y-axis) of IFN-γ signaling, TNF signaling, its related modules respectively and (C) IFNα signaling on samples available from 6 days post symptom onset (PSO) through >60 days PSO (x-axis). PASC participants from the inflammatory clusters 4 and 5 are represented here as inflammatory PASC (red), PASC participants from clusters 2 and 3 are represented here as non-inflammatory PASC (blue) while the recovered participants are represented in black.

Supplementary Figure S11

A

Total number of analytes in Olink panels

AIFI cohort (total = 1463) INCOV cohort (Su et,al) (total = 442)

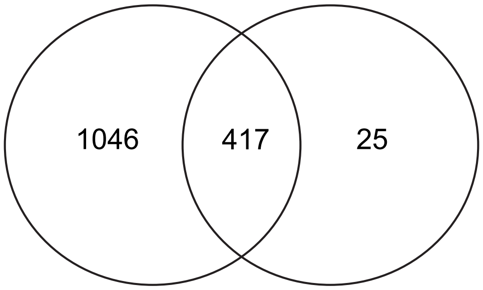

B

Number of AIFI inflammatory markers overlap

AIFI cohort inflammatory markers (510) INCOV cohort (Su et,al) (total analytes = 442)

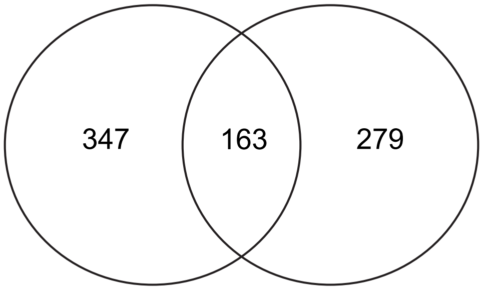

Figure S11. Overlap of in-house and test dataset Olink proteins. (A) Venn diagram of the overlap of total number of Olink panel analytes measured between our (Allen Institute for Immunology - AIFI) cohort and the INCOV (Su et al) cohort. (B) Venn diagram of the overlap of our inflammatory PASC markers with the Su et al cohort.

Supplementary Figure S12

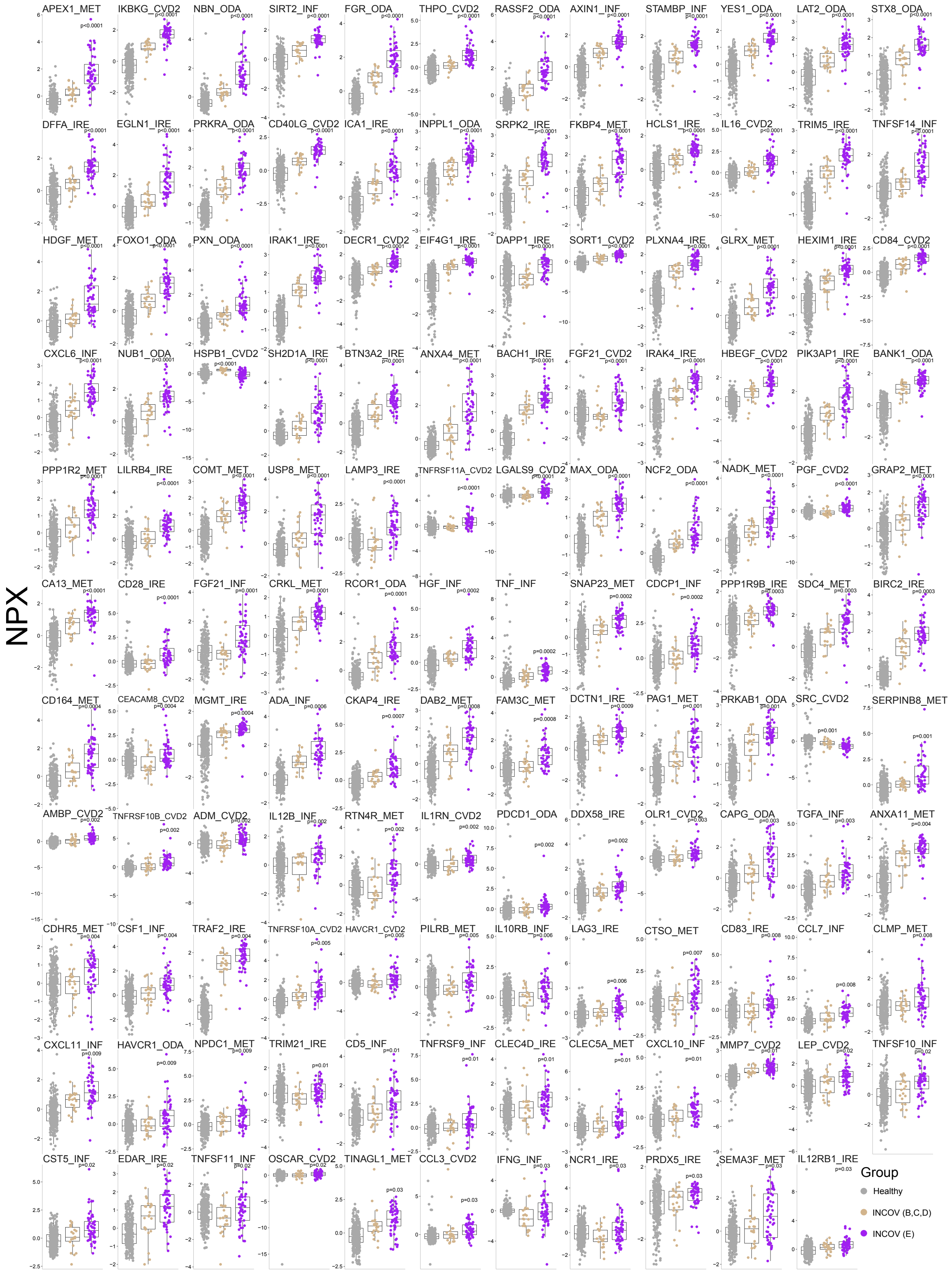

Figure S12. Proteins differentially expressed between the inflammatory and non-inflammatory INCOV clusters. Box plots of the proteins significantly upregulated in the INCOV participants in cluster E (n=53) vs INCOV participants in clusters B,C,D (n=22). All uninfected healthy controls (n=289) are also shown. The y-axis represents the Olink Normalized Protein Expression (NPX). P-values were determined by a two-sided Wilcoxon test. All box plots show the median (centerline), the first and third quartiles (the lower and upper bound of the box) and the whiskers show the 1.5x interquartile range of the data.

Supplementary Figure S13

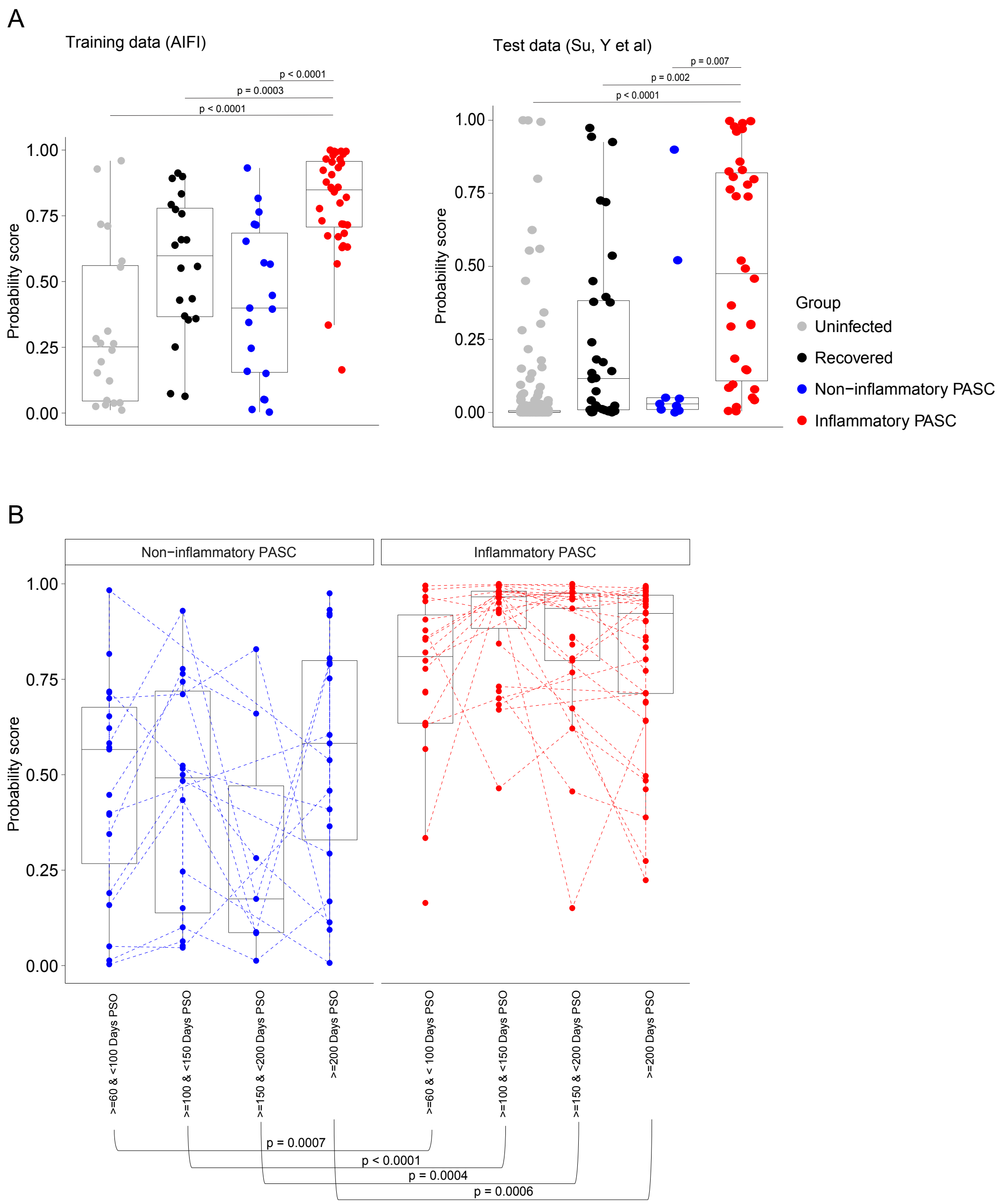

Figure S13. A three-marker protein panel distinguishing inflammatory versus non-inflammatory PASC. Boxplots of the probability score of a logistic regression (LogReg) model of three proteins (CCL7, CD40LG and S100A12) for distinguishing inflammatory versus non-inflammatory PASC on the training data (left panel, our PASC cohort, n=20 uninfected, n=20 recovered, n=19 non-inflammatory PASC, n=36 inflammatory PASC) and the test data (right panel, INCOV: n=289 uninfected, n=32 recovered, n=9 non-inflammatory PASC, n=34 inflammatory PASC). P-values were determined by a two-sided Wilcoxon test. (B) Boxplots of the LogReg probability score on all longitudinal time points on the training data. Each participant with longitudinal time points is connected by dotted lines. The p-values determined by a two-sided Wilcoxon test were compared between inflammatory VS non -inflammatory PASC within each day's post symptom onset (PSO) bin. All box plots show the median (centerline), the first and third quartiles (the lower and upper bound of the box) and the whiskers show the 1.5x interquartile range of the data.

Supplementary Figure S14

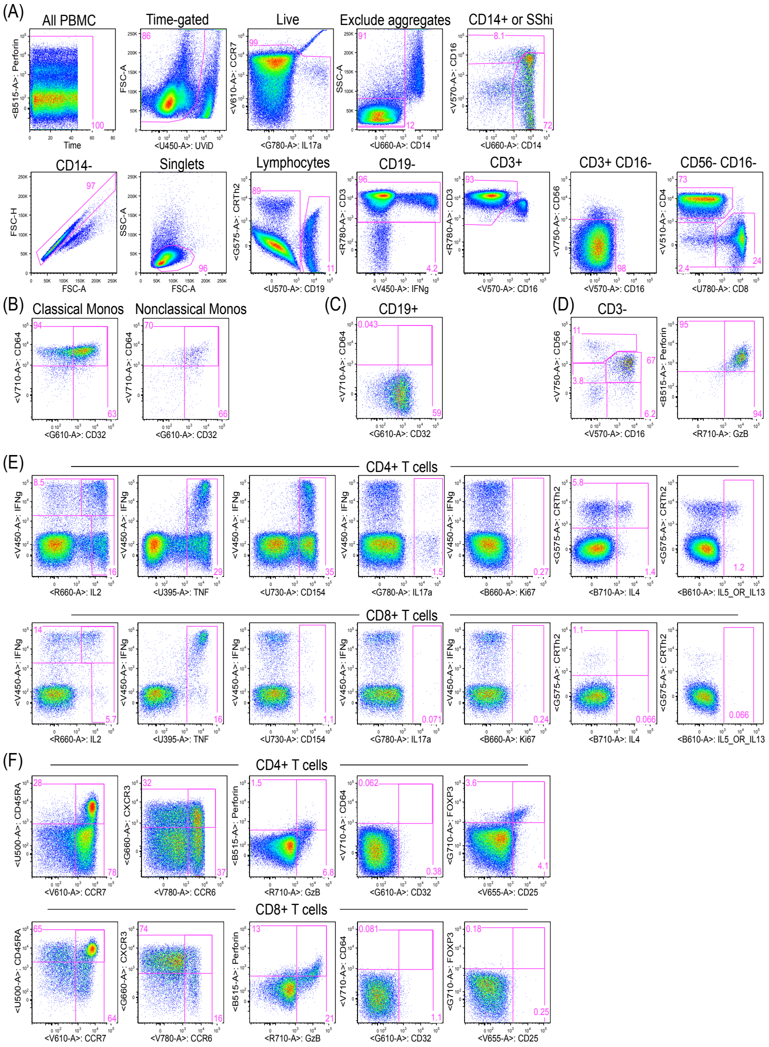

Figure S14. Gating strategy used for flow cytometry data. Previously cryopreserved PBMC from a healthy adult donor were stimulated with staphylococcal enterotoxin B (SEB) for 6 hours as a positive control, stained, and the data were subsequently acquired on a BD FACSymphony instrument. (A) Gating hierarchy to identify lineages. Initial gating on time (seconds) to exclude any events early in collection due to pressure fluctuations, live cell gating, exclusion of aggregates. Monocytes are gated as the inverse of the CD14-SSlo gate, and the upper right graph shows two monocyte subsets based on CD14 vs. CD16. Non-monocytes are gated as CD14-, followed by singlets, and scatter gated on lymphocytes. CD19+ cells are gated against CRTh2 due to the extreme spread of the BUV563 reagent into the G575 detector. CD3+/CD3- cells are gated against IFN-γ to ensure that any CD3+ cells that have downregulated expression during stimulation are captured. T cells are further defined as CD3+ CD16- on a CD3 vs CD16 plot followed by gating out CD56+ NK T cells on a CD56 vs CD16 plot. The T cells are further defined by CD4 or CD8 expression on a CD4 vs CD8 plot. (B) CD32 vs. CD64 expression of monocyte subsets. (C) CD32 vs. CD64 expression on CD19+ B cells. (D) NK cell subsets defined by CD16 vs. CD56 or Perforin vs. Granzyme B on CD3- lymphocytes. (E) Functional markers for CD4+ and CD8+ T cells. A gate is applied for each cytokine and Boolean gates are created to identify cells expressing different combinations of markers. Most gates are copied, applied to all lineages, and cloned so that any changes to the gate on one lineage changes the gate on all lineages. (F) Additional functional and non-functional markers for CD4 and CD8 T cells. Participant PBMC were stimulated with peptide pools as describe in Methods and gated using the same strategy.

Supplementary Table S1

Group	Uninfected	Infected Recovered	Infected PASC
# of Participants	22	24	55
**Demographics**			
Sex			
Female	10 (45.5%)	15 (62.5%)	34 (61.8%)
Male	12 (54.5%)	9 (37.5%)	21 (38.2%)
Age in years – Median (range)			
Female	44 (29-61)	46 (20-79)	52 (22-74)
Male	52 (31-77)	56 (24-65)	47 (31-82)
**Symptoms >60 days PSO**			
Fever & Chills			3 (5.5%)
Loss of Appetite or Weight Loss			2 (3.6%)
Fatigue/Malaise			25 (45.5%)
Pulmonary			23 (41.8%)
Cardiovascular			8 (14.5%)
Gastrointestinal			3 (5.5%)
Musculoskeletal			9 (16.4%)
Neurologic			15 (27.3%)
Tinnitus			2 (3.6%)
Mild Symptoms (any)			35 (63.6%)

Table S1. Study cohort, demographics, and proportion of previously infected study participants exhibiting particular PASC symptoms at ≥60 days post symptom onset (PSO).
