# Supplementary material for: Persistent serum protein signatures define an inflammatory subcategory of long COVID
Source: Nat Commun. 2023 Jun 9;14:3417. doi: 10.1038/s41467-023-38682-4 (PMC10252177; doi:10.1038/s41467-023-38682-4)
Supplement: Supplementary file 2 — Description of Additional Supplementary Files [file 41467_2023_38682_MOESM2_ESM.pdf]

Description of Additional Supplementary Files

File Name: Supplementary Data 1
Description: Sample demographics and clinical metadata of all uninfected, infected recovered and infected PASC participants.

File Name: Supplementary Data 2
Description: Normalized Protein Expression (NPX) matrix of the serum Olink explore panel per sample.

File Name: Supplementary Data 3
Description: List of differential expressed proteins between all uninfected, infected recovered and infected PASC participants (at their first time point available ≥60-days post symptom onset). The p-values of proteins were determined by comparing expression of a protein in one group to the expression of the same protein in all other groups using a two-sided Wilcoxon test and adjusted for multiple comparisons using the Benjamini and Hochberg (BH) method.

File Name: Supplementary Data 4
Description: Table of the 85 rule-in based pathways that were collapsed into 54 modules identified via the enrichment map strategy. The gene memberships of each module and their occurrence in each geneset is listed

File Name: Supplementary Data 5
Description: Table of the intersection of protein markers reported in obesity and aging with the inflammatory PASC signature.

File Name: Supplementary Data 6
Description: The Single Sample Gene Set Enrichment Analysis (ssGSEA) score per sample of the 54 modules identified via the rule-in approach.

File Name: Supplementary Data 7
Description: Table of the modules significantly differentially expressed between clusters identified via the rule-in approach. The p-values were determined by comparing the expression of a module in one cluster of participants to the expression of the same module in all other clusters using a two-sided Wilcoxon test and adjusted for multiple comparisons using the Benjamini and Hochberg (BH) method.

File Name: Supplementary Data 8
Description: Table of the proteins significantly differentially expressed between clusters identified via the rule-in approach. The p-values were determined by comparing expression of a protein in one cluster of participants to the expression of the same protein in all other clusters using a two-sided Wilcoxon test and adjusted for multiple comparisons using the Benjamini and Hochberg (BH) method.

File Name: Supplementary Data 9
Description: Table of the proteins significantly differentially expressed between infected participants in INCOV cluster E and infected participants in INCOV clusters B,C,D combined. The p-values were determined using a two-sided Wilcoxon test and adjusted for multiple comparisons using the Benjamini and Hochberg (BH) method.

File Name: Supplementary Data 10
Description: Table of the fifteen proteins selected as candidates for a panel to distinguish inflammatory versus non-inflammatory PASC. The adjusted p-value of the protein that determined the inflammatory PASC cluster, determined by a two-sided Wilcoxon test, by comparing each cluster to all other clusters and adjusted for multiple comparisons is provided, followed by the logistic regression coefficients and its p-value for the three best proteins that distinguished inflammatory versus non-inflammatory PASC.

File Name: Supplementary Data 11
Description: Table of antibodies and reagents used for the Intracellular Cytokine Staining (ICS) assay.
