# Supplementary material for: Persistent serum protein signatures define an inflammatory subcategory of long COVID
Source: Nat Commun. 2023 Jun 9;14:3417. doi: 10.1038/s41467-023-38682-4 (PMC10252177; doi:10.1038/s41467-023-38682-4)
Supplement: Supplementary file 14 — Reporting Summary [file 41467_2023_38682_MOESM14_ESM.pdf]

nature portfolio

Corresponding author(s): Troy R. Torgerson
Last updated by author(s): May 1, 2023

Reporting Summary

Nature Portfolio wishes to improve the reproducibility of the work that we publish. This form provides structure for consistency and transparency in reporting. For further information on Nature Portfolio policies, see our Editorial Policies and the Editorial Policy Checklist.

Statistics

For all statistical analyses, confirm that the following items are present in the figure legend, table legend, main text, or Methods section.

n/a	Confirmed	
☐	☒	The exact sample size (*n*) for each experimental group/condition, given as a discrete number and unit of measurement
☐	☒	A statement on whether measurements were taken from distinct samples or whether the same sample was measured repeatedly
☐	☒	The statistical test(s) used AND whether they are one- or two-sided<br>*Only common tests should be described solely by name; describe more complex techniques in the Methods section.*
☐	☒	A description of all covariates tested
☐	☒	A description of any assumptions or corrections, such as tests of normality and adjustment for multiple comparisons
☐	☒	A full description of the statistical parameters including central tendency (e.g. means) or other basic estimates (e.g. regression coefficient) AND variation (e.g. standard deviation) or associated estimates of uncertainty (e.g. confidence intervals)
☐	☒	For null hypothesis testing, the test statistic (e.g. *F*, *t*, *r*) with confidence intervals, effect sizes, degrees of freedom and *P* value noted<br>*Give P values as exact values whenever suitable.*
☒	☐	For Bayesian analysis, information on the choice of priors and Markov chain Monte Carlo settings
☒	☐	For hierarchical and complex designs, identification of the appropriate level for tests and full reporting of outcomes
☐	☒	Estimates of effect sizes (e.g. Cohen's *d*, Pearson's *r*), indicating how they were calculated

Our web collection on statistics for biologists contains articles on many of the points above.

Software and code

Policy information about availability of computer code

Data collection	Olink proteomic analysis was performed by Olink and initial data processing performed using the proprietary Olink MyData cloud software package.
Data analysis	Data analysis was performed using packages and functions in R-Studio version 4.1. The packages ggplot2 v3.4.0, pROC v1.18.0, cluster v2.1.4, nlme v3.1-152, mcapply v3.4.1, factoextra v1.0.7, ComplexHeatmap v2.10.0, igraph v1.3.5 and GSVA v1.40 were used for data analysis and visualizations. All figure panels were assembled using Adobe Illustrator (version 27.2; https://www.adobe.com/products/illustrator.html). R Code used to perform analysis and generate figures along with source data files are made available at Zenodo: https://doi.org/10.5281/zenodo.7872791 and GitHub: https://github.com/aifimmunology/PASC-proteomics-talla-vasaikar-et-al.

For manuscripts utilizing custom algorithms or software that are central to the research but not yet described in published literature, software must be made available to editors and reviewers. We strongly encourage code deposition in a community repository (e.g. GitHub). See the Nature Portfolio guidelines for submitting code & software for further information.

Data

Policy information about availability of data

All manuscripts must include a data availability statement. This statement should provide the following information, where applicable:

- Accession codes, unique identifiers, or web links for publicly available datasets
- A description of any restrictions on data availability
- For clinical datasets or third party data, please ensure that the statement adheres to our policy

The processed Olink data generated in this study, the processed Su Y et al (2022) INCOV Olink data, the sample metadata for both studies, the input canonical genesets database, "c2.cp.v7.2.symbols", from MsigDB (v7.2) have been deposited in Zenodo: https://doi.org/10.5281/zenodo.7872791 and GitHub: https://github.com/aifimmunology/PASC-proteomics-talla-vasaikar-et-al. The processed Olink data generated in this study is also provided in the supplementary data files, Supplementary Table S2 and Supplementary Table S6.

Human research participants

Policy information about studies involving human research participants and Sex and Gender in Research.

Reporting on sex and gender	Sex is reported for all participants. Sex of participants was determined based on self-reporting. Patients were enrolled based on symptoms and were not excluded based on sex or age. In the end, subjects were well balanced for sex and had a range of ages. Sex or gender analysis was not performed. Informed consent was obtained from all participants at the Seattle Vaccine Trials Unit and to publish individual's indirect identifiers such as exact age, sex, and BMI. The Fred Hutchinson Cancer Research Center Institutional Review Board approved the studies and procedures.
Population characteristics	Participants were enrolled in the greater Seattle area in the U.S, They were adults at risk for SARS-CoV2 infection or diagnosed with SARS-CoV-2 by a commercially available PCR assay run in a CLIA-approved lab. The study cohort consisted of 55 adults (21 men, 34 women; age 22-82 years) with persistent symptoms lasting ≥60 days after an acute, PCR-confirmed SARS-CoV-2 infection (termed "PASC"), 24 (9 men, 15 women; age 20-79 years) who symptomatically recovered after a PCR-confirmed SARS-CoV-2 infection (termed "Recovered"), and 22 (12 men, 10 women; age 29-77) who had a negative nasopharyngeal PCR test (termed "Uninfected"). Participants were enrolled during the ancestral strain infection of the COVID-19 pandemic. The majority of participants had mild COVID symptoms during their acute SARS-CoV-2 infection (World Health Organization (WHO) ordinal scale 2 or 3). Only 3 participants were hospitalized and required oxygen therapy (WHO ordinal scale 5). Two of these received Remdesivir. One additional participant received steroids. No participants in this cohort required mechanical ventilation or underwent chest computed tomography (CT). All were unvaccinated at the time of enrollment.
Recruitment	Persons at high-risk for a SARS-CoV-2 Infection or persons that exhibited clinical symptoms were recruited from the greater Seattle area. Eligibility criteria included individuals age 18 or over either at high-risk for SARS-CoV2 infection or diagnosed with SARS-CoV-2 infection by a commercially available PCR assay. Challenges associated with recruitment and sample collection, adapting research priorities, and supply-chain difficulties during the global COVID-19 pandemic required that samples be collected in two batches that impacted enrollment and batch design. Participants were not compensated for being in this study. The pilot cohort was collected early in the pandemic (April 2020) with the aim of studying the difference between SARS-CoV-2 infected (n=15) versus uninfected donors (n=22). Two of the fifteen infected donors developed PASC and had persistent inflammatory signatures over time, which prompted a larger study specifically focused on PASC. Fifty-three additional PASC donors and 11 recovered COVID-19 donors were added. Hence, the two batches have a bias on disease status (PASC, recovered, and uninfected; $p<0.05$) but no biases on biological sex or age ($p>0.05$) were observed.
Ethics oversight	Informed consent was obtained from all participants at the Seattle Vaccine Trials Unit and the Fred Hutchinson Cancer Research Center Institutional Review Board approved the studies and procedures.

Note that full information on the approval of the study protocol must also be provided in the manuscript.

Field-specific reporting

Please select the one below that is the best fit for your research. If you are not sure, read the appropriate sections before making your selection.

☒ Life sciences ☐ Behavioural & social sciences ☐ Ecological, evolutionary & environmental sciences

For a reference copy of the document with all sections, see nature.com/documents/nr-reporting-summary-flat.pdf

Life sciences study design

All studies must disclose on these points even when the disclosure is negative.

Sample size	No pre-determined sample size was performed prior to the start of study since patients were enrolled based on self-identification as being at high-risk for infection or having symptoms. Enrolled patients were followed prospectively.

Data exclusions	No data was excluded from the analyses.
Replication	Although replicate samples collected at the same timepoint, were not included in this study, we did perform assays in longitudinal samples collected from participants to assess stability or persistence of signatures over time.
Randomization	Randomization was not relevant to this study. Challenges associated with recruitment and sample collection, adapting research priorities, and supply-chain difficulties during the global COVID-19 pandemic required that samples be collected in two batches between 2020 and 2021, which impacted batch design. The first batch included samples collected early in the pandemic (April 2020) and the second batch included samples collected after this timepoint. As this was an observational trial and not a therapeutic trial, patients were not randomized. To allow us to mitigate potential batch effects, we used 42 cohort samples as bridging controls for batch correction between the two batches. We assessed data pre and post batch correction to ensure that technical variation due to batch are mitigated before performing any analyses.
Blinding	This was an observational study that was not blinded. Patients at high-risk for being infected with SARS-CoV-2 and patients with suspected infection were enrolled in the study and followed prospectively. SARS-CoV-2 infection was confirmed by CLIA-approved PCR testing.

Reporting for specific materials, systems and methods

We require information from authors about some types of materials, experimental systems and methods used in many studies. Here, indicate whether each material, system or method listed is relevant to your study. If you are not sure if a list item applies to your research, read the appropriate section before selecting a response.

Materials & experimental systems

n/a	Involved in the study
☐	☒ Antibodies
☒	☐ Eukaryotic cell lines
☒	☐ Palaeontology and archaeology
☒	☐ Animals and other organisms
☐	☒ Clinical data
☒	☐ Dual use research of concern

Methods

n/a	Involved in the study
☒	☐ ChIP-seq
☐	☒ Flow cytometry
☒	☐ MRI-based neuroimaging

Antibodies

Antibodies used

The Specificity, Fluorochrome, Clone, Vendor, Catalogue, Titer (ul of reagent per 50ul staining volume), Staining Time and Staining Step respectively, for each antibody are listed below:

Perforin: FITC, B-D48, BioLegend, 353310, 1.0, 30, Intracellular
IL-5: BB630, TRFK5, BD, Custom, 0.13, 30, Intracellular
IL-13: BB630, JES10-5A2, BD, Custom, 0.5, 30, Intracellular
Ki67: BB660, B56, BD, Custom, 0.25, 30, Intracellular
IL-4: BB700, MP4-25D2, BD, Custom, 0.05, 30, Intracellular
CRTh2: PE, BM16, BioLegend, 350106, 2.5, 20, Surface
CD32: PE-Dazzle594, FUN-2, BioLegend, 303218, 0.01, 20, Surface
CXCR3 (CD183): PE-Cy5, 1C6/CXCR3, BD, 551128, 10.0, 20, Surface
FOXP3: PE-Cy5.5, PCH101, Invitrogen, 35-4776-42, 1.0, 30, Intracellular
IL-17a: PE-Cy7, BL168, BioLegend, 512315, 0.06, 30, Intracellular
IL-2: APC, MQ1-17H12, BioLegend, 500310, 0.25, 30, Intracellular
Granzyme B: Alexa 700, GB11, BD, 560213, 0.25, 30, Intracellular
CD3: APC-Fire750, UCHT1, BioLegend, 300470, 0.1, 30, Intracellular
TNF: BUV395, MAb11, BD, 563996, 0.1, 30, Intracellular
Viability: UViD, N/A, Invitrogen, N/A, 0.2, 20, Surface
CD45RA: BUV496, HI100, BD, 750258, 0.03, 20, Surface
CD19: BUV563, SJ25C1, BD, 612916, 0.5, 20, Surface
CD14: BUV661, MtP9, BD, 741684, 0.02, 20, Surface
CD154: BUV737, TRAP1, BD, 748983, 0.2, 30, Intracellular
CD8: BUV805, SK1, BD, 612889, 0.15, 30, Intracellular
IFNg: V450, B27, BD, 560371, 1.0, 30, Intracellular
CD4: BV480, SK3, BD, 566104, 0.2, 30, Intracellular
CD16: BV570, 3G8, BioLegend, 302036, 0.25, 20, Surface
CCR7: BV605, G034H7, BioLegend, 353224, 5.0, 20, Surface
CD25: BV650, M-A251, BD, 563719, 2.5, 20, Surface
CD64: BV711, 10.1, BioLegend, 305042, 0.5, 20, Surface
CD56: BV750, 5.1H11, BioLegend, 362556, 0.5, 20, Surface
CCR6 (CD196): BV786, 11A9, BD, 563704, 5.0, 20, Surface

Validation

All antibodies are commercially available and validated by the vendors. No additional validation was performed.

Clinical data

Policy information about clinical studies

All manuscripts should comply with the ICMJE guidelines for publication of clinical research and a completed CONSORT checklist must be included with all submissions.

Clinical trial registration	No intervention, not a clinical trial
Study protocol	As noted above, individuals at high risk for infection or individuals that had COVID symptoms were enrolled in the study. Each participant had a baseline blood draw and were tested for SARS-CoV-2 viral infection by a CLIA PCR assay. Individuals with a positive PCR were followed prospectively and samples were collected at future clinic visits.
Data collection	Mobile COVID testing and phlebotomy clinics were setup at fire stations across the greater Seattle,Washington area. Individuals at high-risk for infection or individuals that were experiencing symptoms were enrolled in the study. Those with a positive CLIA SARS-CoV-2 PCR were followed longitudinally through convalescence and into the long-COVID period. Serum samples were chilled, processed, and frozen within 4 hours of blood draw. Samples were submitted to Olink for testing.
Outcomes	Olink serum proteomic data was used to identify a subset of Long COVID participants that have evidence of persistent inflammation based on expression of cytokines, chemokines, and other inflammatory markers. One subset of participants had a dominant signature of IFN-gamma, associated inflammatory cytokines and chemokines and one subset had evidence of ongoing neutrophil activation. Serum protein signatures were correlated to persistent symptom activity as outlined in the paper.

Flow Cytometry

Plots

Confirm that:

☒ The axis labels state the marker and fluorochrome used (e.g. CD4-FITC).

☒ The axis scales are clearly visible. Include numbers along axes only for bottom left plot of group (a 'group' is an analysis of identical markers).

☒ All plots are contour plots with outliers or pseudocolor plots.

☒ A numerical value for number of cells or percentage (with statistics) is provided.

Methodology

Sample preparation	Cryopreserved PBMC from a healthy adult donor were stimulated with staphylococcal enterotoxin B (SEB) for 6 hours and participant PBMC were stimulated with various peptide pools as described in the methods. Cells were stained, washed, analyzed, and gated according to the attached figure showing the gating strategy.
Instrument	BD FACSymphony
Software	Data acquisition on instrument: BD FACS Diva software. Data analysis: FlowJo, v9.4
Cell population abundance	Analysis was performed on unsorted PBMC, no cell populations were sorted.
Gating strategy	Initial gating on time (seconds) to exclude any events early in collection due to pressure fluctuations, live cell gating, exclusion of aggregates. Monocytes are gated as the inverse of the CD14-SSlo gate, and the upper right graph shows two monocyte subsets based on CD14 vs. CD16. Non-monocytes are gated as CD14-, followed by singlets, and scatter gated on lymphocytes. CD19+ cells are gated against CRTh2 due to the extreme spread of the BUV563 reagent into the G575 detector. CD3+/CD3- cells are gated against IFN-γ to ensure that any CD3+ cells that have downregulated expression during stimulation are captured. T cells are further defined as CD3+ CD16- on a CD3 vs CD16 plot followed by gating out CD56+ NK T cells on a CD56 vs CD16 plot. The T cells are further defined by CD4 or CD8 expression on a CD4 vs CD8 plot. CD32 vs. CD64 expression of monocyte subsets. CD32 vs. CD64 expression on CD19+ B cells. NK cell subsets defined by CD16 vs. CD56 or Perforin vs. Granzyme B on CD3- lymphocytes. Functional markers for CD4+ and CD8+ T cells - A gate is applied for each cytokine and Boolean gates are created to identify cells expressing different combinations of markers. Most gates are copied, applied to all lineages, and cloned so that any changes to the gate on one lineage changes the gate on all lineages.

☒ Tick this box to confirm that a figure exemplifying the gating strategy is provided in the Supplementary Information.
